# Structural Characterization and Profiles of Saponins from Two Algerian Sea Cucumbers

**DOI:** 10.3390/molecules29225346

**Published:** 2024-11-13

**Authors:** Ihcene Khodja, Karim Mezali, Philippe Savarino, Pascal Gerbaux, Patrick Flammang, Guillaume Caulier

**Affiliations:** 1 Protection, Valorization of Coastal Marine Resources and Molecular Systematics Laboratory, Department of Marine Science and Aquaculture, Faculty of Natural Sciences and Life, Abdelhamid Ibn Badis University—Mostaganem, Route nationale N° 11, P.O. Box 227, Kharrouba, Mostaganem 27000, Algeria; ihcene.khodja.etu@univ-mosta.dz; 2 Organic Synthesis and Mass Spectrometry Laboratory, Research Institute for Biosciences, University of Mons—UMONS, 23, Place du Parc, B-7000 Mons, Belgium; philippe.savarino@outlook.fr; 3 Biology of Marine Organisms and Biomimetics Unit, Research Institute for Biosciences, University of Mons—UMONS, 23, Place du Parc, B-7000 Mons, Belgium; patrick.flammang@umons.ac.be (P.F.); guillaume.caulier@umons.ac.be (G.C.)

**Keywords:** echinoderms, holothurians, sea cucumber, triterpene glycoside, mass spectrometry, saponin profile, chemotaxonomy, Algerian basin

## Abstract

Sea cucumbers are benthic marine invertebrate members of the phylum Echinodermata. Due to the absence of a rigid skeleton, these species have developed chemical defenses based on the production of saponins (triterpene glycosides). These secondary metabolites are bioactive molecules with a broad biological, ecological, and pharmaceutical spectrum. However, the saponin profiles of several species of sea cucumbers are not known yet. The present study aims to highlight the mixture of saponins in two sea cucumber species from the Algerian coast, namely *Holothuria* (*Holothuria*) *algeriensis*, which has been recently described in central and western Algerian waters, and *Holothuria* (*Roweothuria*) *arguinensis*, originating from the Atlantic Ocean and reported in Algeria for the first time in 2014. Saponin extracts from three individuals of *H. (H.) algeriensis* and two individuals of *H. (R.) arguinensis* were analyzed using mass spectrometry, i.e., Matrix-assisted Laser Desorption/Ionization mass spectrometry (MALDI-MS), MALDI-High Resolution MS (MALDI-HRMS), Liquid Chromatography MS (LC-MS) and tandem MS (LC-MS/MS). These analyses allow us to detect 11 and 18 elemental compositions for *H. (H.) algeriensis* and *H. (R.) arguinensis*, respectively, each presenting several isomers. In total, 13 new saponin structures are proposed, of which four are common between the two species, six are specific to *H. (H.) algeriensis* and three to *H. (R.) arguinensis*. The saponin profiles of the two species were compared to those of other species of the same genus existing on the Algerian coast and the results showed that they share non-sulfated saponins with *Holothuria* (*Panningothuria*) *forskali* and *Holothuria* (*Platyperona*) *sanctori* and sulfated saponins with *Holothuria* (*Holothuria*) *tubulosa* and *Holothuria* (*Roweothuria*) *poli*.

## 1. Introduction

Marine organisms are a valuable source of high-quality food and represent novel reservoirs of biologically active components [1,2,3]. Sea cucumbers are benthic invertebrates that play important ecological roles such as bioturbation, sediment cleaning, and organic matter recycling [4,5,6]. These organisms also have interesting nutritional and medicinal values [7,8]. They are a precious source of high-quality proteins, healthy lipids, vitamins, and minerals for the human diet, and their use as food or in traditional medicine started about 1000 years ago in Asia and the Middle East [9]. The biological and pharmacological activities exhibited by these organisms (anti-angiogenic, anticancer, anticoagulant, anti-inflammatory, antimicrobial...) [10,11,12,13,14,15] could be attributed to the presence of appreciable amounts of bioactive compounds, including saponins [16,17,18]. The amphiphilic properties of the latter enable them to interact with cell membranes as surface-active molecules that may, ultimately, disrupt the membrane [18,19].

Saponins are amphiphilic compounds composed of two parts: the aglycone moiety (lipophilic and liposoluble) and the glycan (hydrophilic and water-soluble) [20,21,22] associated via a glycosidic bond [23]. According to the nature of the aglycone, also called genin or sapogenin, saponins are divided into three major classes: triterpene, steroidal and steroidal alkaloid glycosides [24]. Those found in sea cucumbers are triterpene aglycones that contain 30 carbon atoms [25]. They are of two types; holostane with a structural feature 3β-hydroxy-5α-lanostano-γ(18,20)-lactone and non-holostane with no γ(18,20)-lactone [20,26]. The glycosidic part can contain up to six monosaccharide units covalently linked to the C-3 of the aglycone. The sugar moieties mainly consist of D-xylose (Xyl), D-quinovose (Qui), D-glucose (Glc), 3-*O*-methyl-D-glucose (MeGlc), 3-*O*-methyl-D-xylose (MeXyl) and sometimes 3-*O*-methyl-D-quinovose (MeQui), 3-*O*-methyl-D-glucuronic acid (MeGlcA) and 6-*O*-acetyl-D-glucose (AcGlc) [20,22,27]. Sixty percent of triterpene glycosides isolated to date from sea cucumbers have sulfate groups attached to the monosaccharide units of the carbohydrate chain. Most of them are monosulfated, but many di- and trisulfated glycosides have also been isolated [20].

Sea cucumber saponins will provide great economic value and application prospects for the clinical medicine, pharmaceutical, and functional health food industry due to various biological activities [23] including antitumor, antifungal, antiviral, hemolytic, cytotoxic, ameliorating hyperlipidemia, fatty liver, limiting fat accumulation, regulating glycemia, preventing gout, relieving hyperuricemia, and promoting bone marrow hematopoietic function [23,28]. For example, a study of *Apostichopus japonicus* (Selenka, 1867) ovary extract demonstrated that it significantly reduces body weight, serum lipids, adipose tissue, and liver weight in mice fed a high-fat diet [29]. Another study has shown that frondoside A is a potential compound that targets multiple characteristics of cancer cells and is used in cancer treatment by inhibiting cancer cell growth, migration, invasion, metastasis, and angiogenesis [30]. Frondoside A treatments also target cancer cells, including pancreatic, lung, colon, and prostate cancer cell lines [30]. Saponins extracted from *Holothuria (Selenkothuria) moebii* Ludwig, 1883 have shown activity in suppressing the proliferation of four different glioma cells [31].

Saponins have several complex ecological functions, the most important being to chemically protect sea cucumbers [32,33]. They are produced in the body wall, viscera, and specific defense organs, such as the Cuvierian tubules [17,34]. In addition, it has been suggested that saponins may contribute to digestion [35], reproduction (spawning synchronization) [36], as well as intra- or interspecific chemical communication [32,37,38].

Each species of sea cucumber contains its own special mixture of saponins, which can be considered unique chemical signatures for envisaging these molecules as chemotaxonomic markers [22,39]. Moreover, saponin mixtures differ qualitatively and quantitatively between species but also according to the body component, sex, and maturity of the individual [16,40,41,42,43]. As far as the Algerian coast is concerned, only the saponins profile of *Holothuria* (*Platyperona*) *sanctori* (Delle Chiaje, 1823) was described confirming its phylogenetic status and its biochemical similarity to *H.* (*Panningothuria*) *forskali* [17].

The present study focuses on two other sea cucumbers species recently reported on the Algerian coast, namely *Holothuria* (*Roweothuria*) *arguinensis* Koehler and Vaney, 1906 and *Holothuria* (*Holothuria*) *algeriensis* Mezali, Thandar and Khodja, 2021. *Holothuria* (*R.*) *arguinensis* is a species that was originally discovered in the Northeast Atlantic Ocean distributed from Portugal to Morocco and Mauritania, including the Canary Islands, associated with sandy/rocky areas and seagrass beds where it is present between 0 and 50 m in depth [44,45,46,47]. However, its geographical distribution has been modified and the species has now been observed in the Mediterranean sea where it has been reported on the Spanish [44] and Algerian coasts [48]. *Holothuria* (*R.*) *arguinensis* does not compete with native Mediterranean sea cucumbers [*Holothuria (Holothuria) tubulosa* (Gmelin, 1791), *Holothuria (Roweothuria) poli* (Delle Chiaje, 1824), *H. (P.) forskali,* and *H. (P.) sanctori*], as it consumes food resources that are rarely used by the other species [49]. This species has reached a high economic value in recent years, being intensively and illegally harvested on the coasts of Algarve and Andalusia [50]. To meet the demand of Asian countries with less impact on wild populations, researchers are trying to improve its aquaculture given its high aquaculture potential and its adaptation to integrated monospecific and multitrophic aquaculture [51,52,53]. Data on *H.* (*H.*) *algeriensis* are scarcely present in the literature since that the species has been only recently separated following a taxonomic revision of the species of the genus *Holothuria* present on the Algerian coast [54]. Prior to this, the species was considered as a morphotype of *H.* (*H.*) *tubulosa* and was referred to as *H.* (*H.*) *tubulosa* “B” in the work of [55]. This species is often found with *H.* (*H.*) *tubulosa* which frequents sandy, muddy and *Posidonia oceanica* Delile, 1813 seagrass biotopes between 0 and 40 m depth [56]. It has been reported in the central and western region of Algeria, but its distribution area is probably more widespread in the Mediterranean sea [48].

The main objective of the present study is to establish for the first time the molecular diversity of saponins extracted from individuals of *H.* (*H.*) *algeriensis* and *H.* (*R.*) *arguinensis* from the Algerian coast and to compare them with those of other genetically related species from the same family.

## 2. Results

### 2.1. General Profile of Saponins

The average dry weight of the analyzed individuals of *H.* (*H.) algeriensis* was 11.81 ± 5.40 g (mean ± SD). The average dry weight of the saponin extract was 21.47 ± 8.90 mg. The saponin concentration calculated for *H.* (*H.*) *algeriensis* amounts thus to a mean value of 1.82 ± 0.75 g/kg. The average dry weight of the saponin extract for *H.* (*R.*) *arguinensis* was 1.35 ± 1.48 mg. However, due to technical issues, the average dry weight of *H.* (*R.*) *arguinensis* could not be measured, preventing us from determining the saponin concentration in this species.

**Figure 1:**
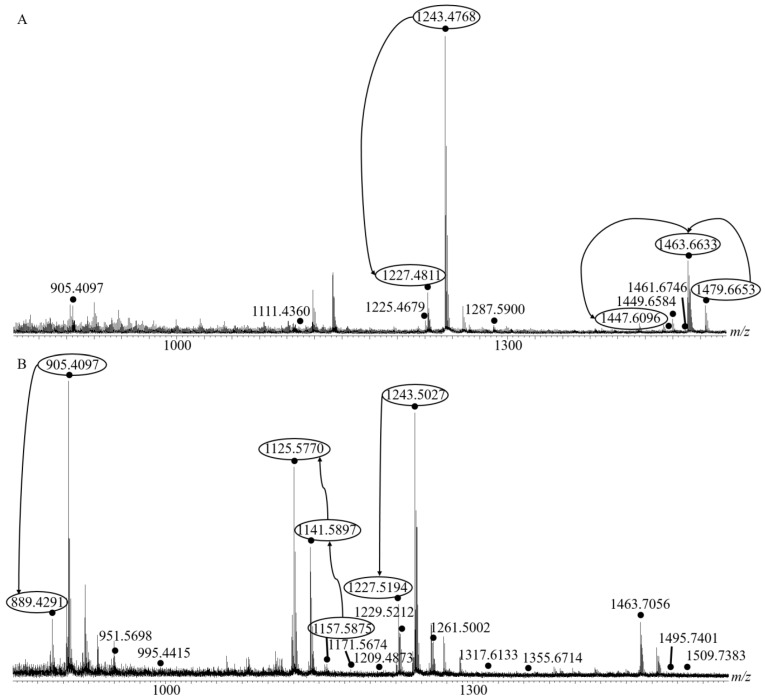
(**A**) Matrix-assisted laser desorption/ionization [MALDI-MS (+)] mass spectrum (recorded with an *m*/*z* external calibration) of the total saponin mixture of *Holothuria* (*H.*) *algeriensis* integument extracts. (**B**) MALDI-MS (+) mass spectrum (recorded with an *m*/*z* external calibration) of the total saponin mixture of *Holothuria* (*R.*) *arguinensis* integument extract. Saponins signals are highlighted by black dots on which the [M+Na]^+^ are noted. The 16 Da mass differences between signals are highlighted by the arrows.

The Matrix-assisted Laser Desorption/Ionization mass spectrum (MALDI-MS) of the saponin extracts obtained for one individual of *H.* (*H.*) *algeriensis* is shown in Figure 1A. Based on the measured mass-to-charge ratios (*m*/*z*), see also Table 1 for accurate mass measurement data (HRMS—High Resolution MS), we detected 11 signals in the *m*/*z* 850–1550 range that potentially correspond to ionized saponins. The other peaks visible in the mass spectrum correspond to fragment ions of saponins generated by the loss of water or sulfate function during MALDI ionization. The three replicates were characterized by similar MALDI-MS profiles, i.e., saponin ions being detected at the same *m*/*z* values. However, the relative intensities between the three spectra were slightly different (Figure S1). In the three cases, the peak corresponding to *m*/*z* 1243 ions was the most intense. It should be noted that some peaks (shown by circles in the mass spectra) could be associated into groups whose signals are separated by mass differences of 16 Da, typically an oxygen atom. The first group gathers the *m*/*z* 1479, 1463, and 1447 ions and the second associates the *m*/*z* 1243 and 1227 ions.

Figure 1B presents the MALDI-MS spectrum of the saponin extract obtained for an individual of *H.* (*R.) arguinensis*. A total of 18 signals in the *m*/*z* 850–1550 range and potentially corresponding to saponin ions was detected. The two replicates show similar MALDI-MS spectra profiles with different relative peak intensities within or between individuals (Figure S2). The most intense peaks were different in the two individuals, corresponding to *m*/*z* 905 and *m*/*z* 1125 in the first and second individual, respectively. Some peaks were associated into groups whose signals are separated by mass differences of 16 Da (typically an oxygen atom). The first group combines the *m*/*z* 1243 and 1227 ions, the second group the *m*/*z* 1157, 1141, and 1125 ions and the third group the *m*/*z* 905 and 889 ions.

The elemental compositions obtained for the saponin ions were confirmed on the basis of accurate mass measurements performed using MALDI-HRMS (High Resolution Mass Spectrometry) as shown in Table 1. This technique revealed that the detected ions possess elemental compositions of 2-sugar sulfated saponins (*m*/*z* 905), 4-sugar non-sulfated saponins (*m*/*z* 1111), 4-sugar sulfated saponins (*m*/*z* 1225–1243), 5-sugar non-sulfated saponins (*m*/*z* 1287), and 6-sugar non-sulfated saponins (*m*/*z* 1447–1479). In total, amongst the 11 detected saponin compositions, four are sulfated (Figure S3; Table 1).

MALDI-HRMS experiments (see Table 2) indicated that the detected ions have elemental compositions of 2-sugar sulfated saponins (*m*/*z* 889–905), 3-sugar non-sulfated saponins (*m*/*z* 951–995), 4-sugar non-sulfated saponins (*m*/*z* 1125–1171), 4-sugar sulfated saponins (*m*/*z* 1209–1261), 5-sugar non-sulfated saponin (*m*/*z* 1317), 5-sugar sulfated saponins (*m*/*z* 1355), and 6-sugar non-sulfated saponins (*m*/*z* 1463–1509). In total, amongst the 18 saponin compositions identified, eight are sulfated (Figure S4; Table 2).

### 2.2. Detection of Saponin Isomers by LC-MS

MALDI-MS data were complemented by liquid chromatography-mass spectrometry (LC-MS) analyses to determine the presence of isomers. As for a typical example, Figure S5 presents the extracted ion current (EIC) chromatogram of the *m*/*z* 1425 ions for the first individual of *H.* (*H.*) *algeriensis* revealing that this saponin composition presents two isomers with retention times at 7.1 min and 7.6 min and proportions of 1.36% and 1.61%, respectively. The isomer molar proportions were estimated from the relative abundances of the ion signals, i.e., by LC signal integration (see experimental section). Figure S6 presents the LC-MS mass spectrum for the specific retention time at 7.6 min featuring abundant *m*/*z* 1425 ions, i.e., [M+H]^+^. We note again that [M+H]^+^ and [M+Na]^+^ ions are mainly generated upon LC-MS and MALDI-MS ionizations, respectively.

For *Holothuria* (*H.*) *algeriensis*, LC-MS analyses revealed the presence of one to three isomers for each elemental composition (Table 1). A total of 21 saponin congeners were detected in the three extracts from the three *H.* (*H.*) *algeriensis* individuals. The molar proportions of each congener were estimated based on MS signal intensities (see experimental section) for the three individuals. For each isomer family, the major proportion is bolded in Table 1. In all the extracts, the congener of the elemental composition C_54_H_86_O_27_S at 7.6 min was the most abundant, followed by that of C_41_H_64_O_17_S at 7.6 min and C_54_H_86_O_26_S at 7.9 min corresponding, respectively, to the signals [M+Na]^+^ at *m*/*z* 1243, 905 and 1227 in the MALDI-ToF spectrum (Figure 1A).

From the LC-MS analysis of the two extracts of *Holothuria* (*R.*) *arguinensis*, the presence, for each elemental composition, of one to three isomers was also demonstrated. A total of 32 saponin congeners were detected in the two extracts (Table 2). In all extracts, the congeners of elemental composition C_41_H_64_O_17_S at 10.0 min was the most abundant, followed by that of C_54_H_86_O_27_S at 7.6 min and C_41_H_64_O_16_S at 10.9 min corresponding, respectively, to the signals [M+Na]^+^ at *m*/*z* 905, 1243, and 889 in the MALDI-ToF spectrum (Figure 1B).

### 2.3. Structural Characterization of Saponins by LC-MS/MS

**Figure 2:**
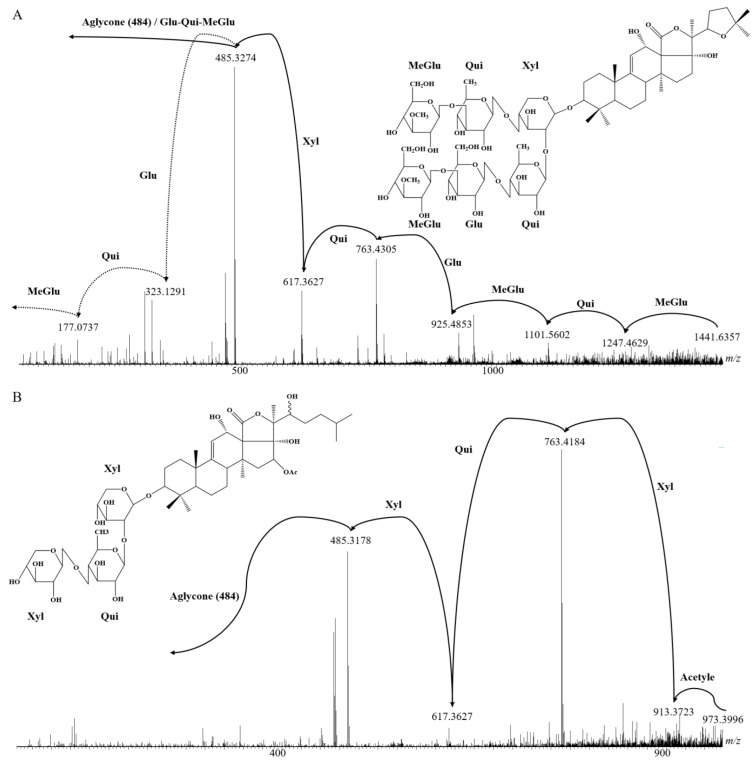
LC-MS/MS analysis of saponin extracts: collision-induced dissociation mass spectra of (**A**) Saponin A [M+H]^+^ ions [=Saponin 8 in *H.* (*R.*) *arguinensis*] and (**B**) Saponin 14 [M+H]^+^ ions identified in *H.* (*R.*) *arguinensis*.

Tandem mass spectrometry (LC-MS/MS) analyses were performed to identify the different saponins either by matching their structure with known saponins or by proposing structures for new congeners based on the fragmentation patterns (see Figure 2 for typical examples).

**Figure 3:**
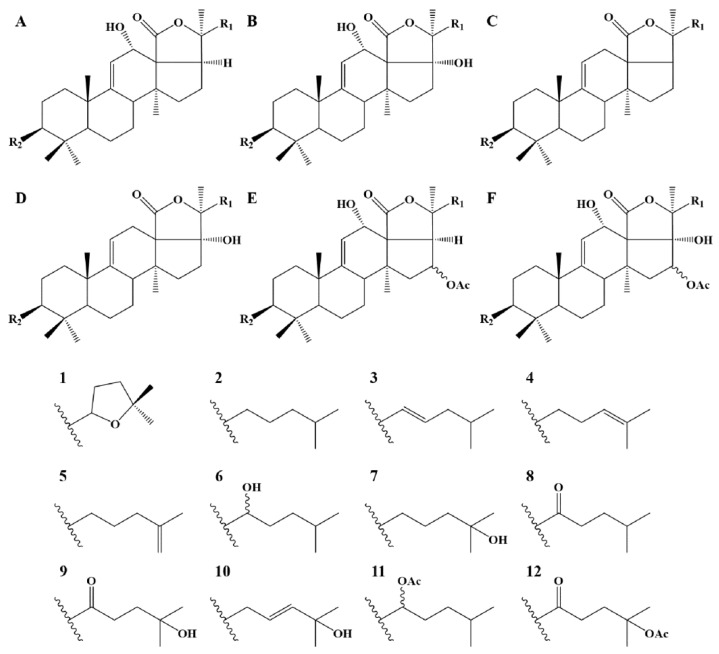
Possible skeletons and side chains of aglycones observed in *Holothuria* (*H.*) *algeriensis* and *Holothuria* (*R.*) *arguinensis*. Capital letters and numbers correspond to those indicated in the “Aglycone-R1” column in Table 3.

**Figure 4:**
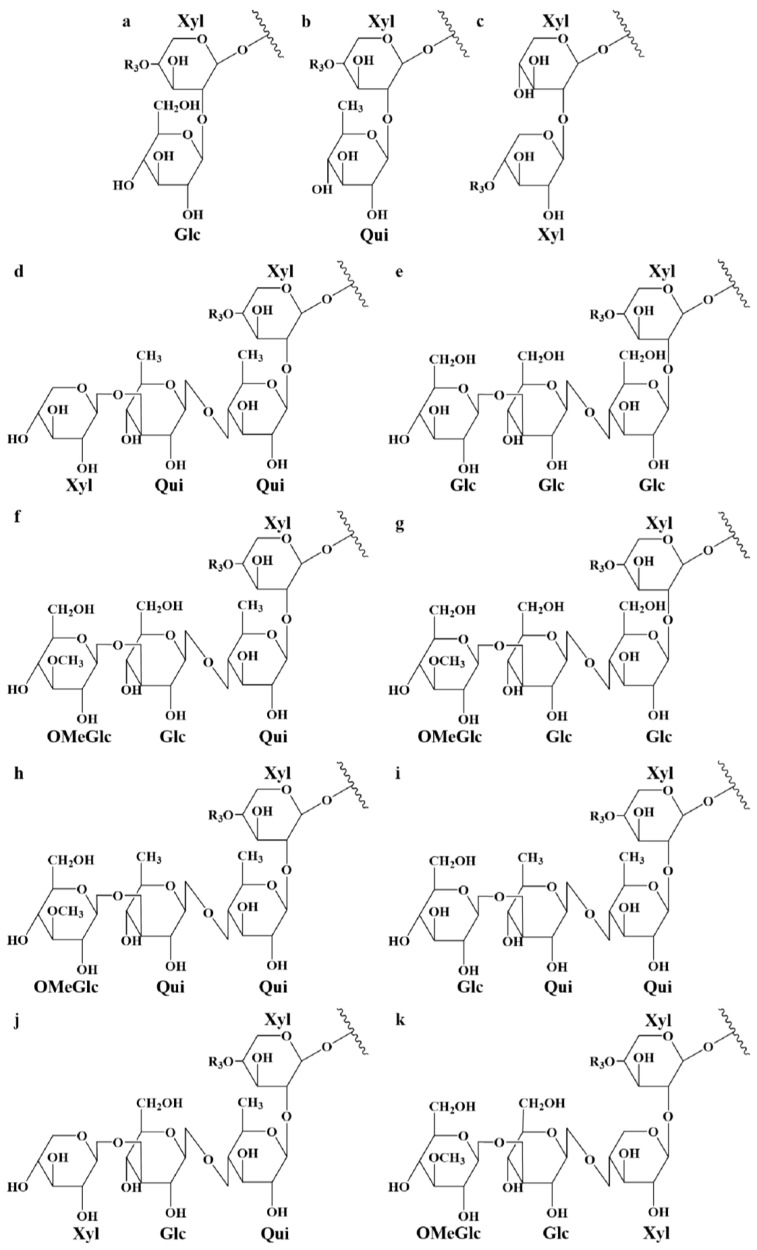
Glycosidic moieties of the saponins identified in extracts of *Holothuria* (*H.*) *algeriensis* and *Holothuria* (*R.*) *arguinensis*. Lowercase letters correspond to those in the “R2” column of Table 3.

**Figure 5:**
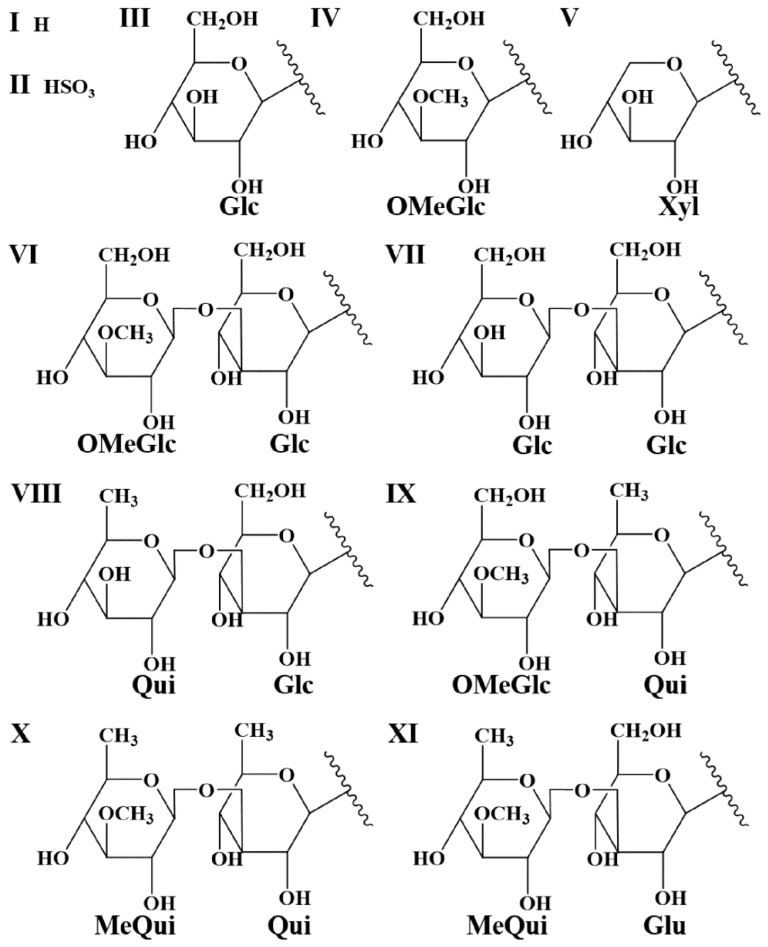
Complementary glycosidic moieties of saponins identified in extracts of *Holothuria* (*H.*) *algeriensis* and *Holothuria* (*R.*) *arguinensis* with three, five and six monosaccharide units. Roman numerals correspond to those in the “R3” column of Table 3.

Whereas MS/MS experiments are quite efficient in establishing the glycan sequence, including the branching (see Figure 2), this method remains insufficient for determining the structure of the aglycone in order to characterize unknown saponins. Saponins need to be purified for individual nuclear magnetic resonance (NMR) analysis. Therefore, in the present investigation, when the fragmentation pattern does not correspond to a saponin reported in the literature, the different possibilities of aglycone structures that correspond to the mass transition are proposed. This is also the case when different saponin structures are matching the MS/MS data. Table 3 summarizes all the detected saponins extracted from *Holothuria* (*H.*) *algeriensis* and *H.* (*R.*) *arguinensis* with their structural characteristics. These are indicated in the table in four different columns, with “Aglycone-R1” indicating the possible structures of the aglycone (Figure 3) as well as the structure of the side chain substituents, “R2” indicating the substituents of the glycosidic moiety corresponding to the two and four monosaccharide units (Figure 4), and “R3” indicating the R2 substituents consisting of one or two monosaccharide units (Figure 5). The new proposed saponins were named “Saponin A” to “Saponin K” for those identified in *H.* (*H.*) *algeriensis* and “Saponin 1” to “Saponin 16” for those identified in *H.* (*R.*) *arguinensis* and are bolded in Table 3.

LC-MS/MS analyses were performed on all the saponin ions [M+H]^+^ for *H.* (*H.*) *algeriensis* detected by MALDI-MS and the characteristics of the different structures are grouped in Table 3. In total, 28 structures were identified for *H.* (*H.*) *algeriensis* of which 11 are new (Figures S7–S15), the total number also includes the different possibilities with different aglycones. The three individuals of *H.* (*H.*) *algeriensis* present three major saponins namely [Scabraside B/25-hydroxyfuscocineroside B/Holothurin A], [Holothurin B/Holothurin B4] at retention times of 7.6 and 10.1 min and Saponin F representing approximately 91.49%, 96.97%, and 92.55% in individuals 1, 2, and 3, respectively, while the other saponins detected represent only 8.51%, 3.03%, and 7.45% for the same individuals mentioned above. Among the structures already identified in other sea cucumber species, five are unique possibilities, i.e., Holothurinosides I at *m*/*z* 1479, G at *m*/*z* 1449, E and O at *m*/*z* 1287 and Nobiliside B (I) at *m*/*z* 905. These saponins are present in weak proportions in individuals 1, 2, and 3 of *H.* (*H.*) *algeriensis* (0.99%, 0.66%, 2.28%, respectively). The structure of these saponins have the aglycone B1 (484 Da), A1 (468 Da) or D1 (452 Da) (in the case of Nobiliside B (I)). Two saponins present the acetyl function, which was highlighted in the LC-MS/MS spectra by differences of 60 Da. These are Saponins H and I (Figures S13 and S14). Back to the MALDI-MS results (see Figure 1), two groups of *m/z* have thus been established with 16 Da differences (Figure 1A). By analyzing the LC-MS/MS spectra, we may conclude that the *m*/*z* couples belonging to the established groups present the same glycosidic chain and that the difference of 16 Da lies in the structure of the aglycone that presents one additional hydroxyl function (an additional oxygen atom). These pairs of saponins correspond to Holothurinoside I and [Holothurinoside H, Marmoratoside B or 17a-hydroxyimpatienside A]; Saponin A and Saponin C; [Scabraside B, 25-hydroxyfuscocineroside B or Holothurin A] and [Scabraside A, 24-dehydroechinoside A, Fuscocineroside C or Fuscocineroside B] and finally Saponin E and Saponin G.

As far as the second sea cucumber species is concerned, LC-MS/MS analysis were performed on the 16 ions detected by MALDI-MS from the saponin extract of *Holothuria* (*R.*) *arguinensis*. In total, due to the detection of numerous isomers, 34 saponin structures were identified of which 16 have, to our knowledge, not yet been described in the literature (Figures S17–S32). Both individuals of *H.* (*R.*) *arguinensis* reveal three dominant saponins, namely [Holothurin B/Holothurin B4] at retention times of 7.6 and 10.1 min, [Scabraside B/25-hydroxyfuscocineroside B/Holothurin A] and [Holothurin B3/24-dehydroechinoside B] representing approximately 89.55% and 91.37% in individuals 1 and 2, respectively. Among the identified structures already listed in the literature, four are readily identified due to unique possibilities, i.e., Holothurinoside N, Desholothurin A, Holothurinoside C and Nobiliside B(I) which correspond to *m*/*z* 1317, 1141, 1125, and 905 ions. These saponins are present in weak proportions in individuals 1 and 2 (2.81% and 3.93%, respectively). These structures also have aglycone structures similar to *H.* (*H.*) *algeriensis*.

Amongst the detected saponin ions, four are common between *H.* (*H.*) *algeriensis* and *H.* (*R.*) *arguinensis* and correspond to *m*/*z* 1441, 1181, 1165, and 843. These are Saponin A (Figure 2A) (Saponin 4), Saponin E (Saponin 6), Saponin F (Saponin 8), and Saponin G (Saponin 9). These saponins are present in higher proportions in *H.* (*H.*) *algeriensis* (between 3.44% and 5.42%) than in *H.* (*R.*) *arguinensis* (1.30% and 1.62%).

MALDI-MS analysis of *H.* (*R.*) *arguinensis* extracts has revealed the presence of three *m*/*z* groups with 16 Da difference between congeners. The saponin couples [Scabraside B/25-hydroxyfuscocineroside B or Holothurin A] and [Scabraside A/24-dehydroechinoside A/Fuscocineroside C or Fuscocineroside B]; Saponin 6 and Saponin 9; Desholothurin A and Holothurinoside C; [Holothurin B or B4] and [Holothurin B3 or 24-dehydroechinoside B] and finally, Nobiliside B (I) and Saponin 16 share the same sugar chain and the difference between them lies in the absence or presence of the hydroxyl function on the aglycone. Out of all the identified structures, three present an acetyl group on the aglycone. As a typical example, the fragmentation scheme and proposed structure of Saponin 14 are illustrated in Figure 2B.

## 3. Discussion

Mass spectrometry analyses, including MALDI-MS, MALDI-HRMS, LC-MS and LC-MS/MS, are relevant methods for the comparison of the saponin contents in different sea cucumber species. Even if it cannot be as accurate in deciphering the molecular structure as Nuclear Magnetic Resonance (NMR) spectroscopy, the different LC-mass spectrometry (MS) techniques used in this study allow us to firstly determine the molecular compositions and then to detect the different isomers for the same composition while affording, in most cases, hypothetical chemical structures [17,57]. This level of chemical investigation allows the biological/ecological/taxonomical interpretations required for marine scientists to understand the evolutionary outcomes of such economies (e.g., [32,37,38]).

### 3.1. Saponins of H. (H.) algeriensis and H. (R.) arguinensis

Analysis of the saponin extracts from both studied species revealed the presence of 11 elemental compositions in *H.* (*H.*) *algeriensis* and 18 in *H.* (*R.*) *arguinensis*, each containing several saponin isomers (Figure 1, Table 1 and Table 2). In addition to the saponin structures already identified in the literature for other species, unreported elemental compositions have been detected in our analyses and could correspond to two or more possibilities of saponins that differ by the non-glycosidic part. Our analyses allowed us to detect 11 and 16 new saponins for *H.* (*H.*) *algeriensis* and *H.* (*R.*) *arguinensis*, respectively, four being common to the two species and detected upon MALDI-MS analysis at *m*/*z* 1463, 1243, 1227 and 905 as [M+Na]^+^ ions. The saponin profile of both species reveals a mixture of non-sulfated and sulfated saponins [four in *H.* (*H.*) *algeriensis* and eight in *H.* (*R.*) *arguinensis*] and of non-acetylated and acetylated saponins [two in *H.* (*H.*) *algeriensis* and seven in *H.* (*R.*) *arguinensis*] (Table 1 and Table 2).

Despite the fact that we did not perform any NMR analysis that would have allowed us to determine the structure of the aglycone, we can still propose some structures for the new saponins. Considering that for the saponins reported in the literature, the aglycones whose mass is 484 Da correspond to structure B1, 468 Da correspond to A1 or D1 (less frequent) and 452 Da correspond to C1, we can propose the structure of the aglycones identified in the two species and propose structures for some of the new saponins identified. Thus, 10 structures are proposed with four being common between both species, namely Saponin A (Saponin 4), Saponin E (Saponin 6), Saponin F (Saponin 8) and Saponin G (Saponin 9); four saponins being detected only in *H.* (*H.*) *algeriensis*, namely Saponin C, D, J, and K and two being present only in *H.* (*R.*) *arguinensis*, namely Saponin 13 and 16 (Table 3).

On the other hand, we can also propose structures for the saponins whose aglycone presents unique possibility, these are Saponins H and I in *H. (H.) algeriensis* and Saponin 14 in *H. (R.) arguinensis* (Table 3, Figures S13, S14 and S30).

For the remaining 16 molecules that could not be strictly identified, six present an aglycone not found in the literature (Saponins 2, 3, 5, 10, 12, and B) and 10 show several aglycone possibilities that differ from each other by the position of the acetyl group in the case of aglycones B11/F2 or the hydroxyl group in the case of aglycones A6/B2 (Table 3).

Individuals of the same species present similar mass spectra in terms of molecular compositions. However, the relative intensities and proportions of each saponin are different between individuals (Figures S1 and S2). These proportions are likely to be influenced by environmental factors and the conditions in which the individuals were before their capture (stress, predation, reproduction, time of the year) [32,37,58]. On the other hand, the difference in proportions between saponins is also influenced by the presence of the sulfate function, as saponins differing only in the presence of this function do not ionize in the same way, so comparison of their relative proportions, respectively, is unreliable.

### 3.2. Comparison with Other Species Belonging to the Genus Holothuria

**Figure 6:**
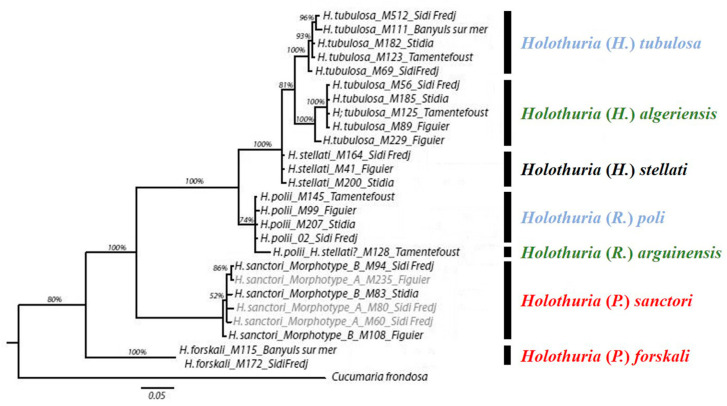
Bayesian consensus tree based on 16S rDNA ([55] modified according to [54]). The values indicated above the branches represent the posterior probabilities (PP in %). Species are colored based on their saponin types: red, non-sulfated; blue, sulfated; green, mixed and black, no available data.

By comparing the saponins content of the investigated species with the other representatives of sea cucumbers belonging to the genus *Holothuria* present on the Algerian coast, we find that they share more saponins with *H.* (*P.*) *sanctori* and *H.* (*P.*) *forskali* than with *H.* (*R.*) *poli* and *H.* (*H.*) *tubulosa*, two species that are phylogenetically closer to them (Figure 6; Table 4) [16,17,32,42,59]. In fact, they share Holothurin A, B, B3 [only in *H.* (*R.*) *arguinensis*] and B4 with *H.* (*R.*) *poli* [59], which belongs to the same subgenus as *H.* (*R.*) *arguinensis*, and Holothurin A and B with *H.* (*H.*) *tubulosa* [59], which belongs to the same subgenus as *H.* (*H.*) *algeriensis* and from which the latter has been recently separated [54]. However, this interpretation should be taken with precaution considering that the results have not been obtained with identical methodologies and mass spectrometers.

Although the studied species share some molecules with other species of the genus *Holothuria* present in the same region (Table 4), their mixtures of saponins remains species-specific, each saponins cocktail being considered as a chemical signature for any sea cucumber species [17,22,37,60]. The role of saponins is far beyond that of a simple toxin, since, in addition to being defensive allomones, they also act as pheromones and interspecific kairomones, suggesting that the evolutionary expansion of their diversity may not have been due solely to their cytotoxic/defensive nature but also to their solubility and potential as species-specific semiochemicals [38]. Thus, saponins represent potential chemo-taxonomic markers of sea cucumbers and reliable models to study [61]. Their biochemical evolution can affect both their fragments, namely the glycone and aglycone [33]. The general trend of glycone evolution in sea cucumbers depends on the presence/absence or number and position of sulfate groups, the type of sugar units and their structure, and the position of the methyl group [22,33] while those of aglycones is more complicated and depends on the presence or absence of lactone, keto, hydroxyl groups as well as the position of double bonds which leads from poorly oxidized to more oxidized compounds [22,61]. The reliability of chemotaxonomy in sea cucumber is caused by the independent control of different structural features of glycosides by a complex set of genes that are independent from each other [61].

Mapping known saponin signatures according to whether they were purely non-sulfated, sulfated, or mixed on a phylogenetic tree of species of the genus *Holothuria* suggest that species with exclusively non-sulfated saponins such as *H.* (*P.*) *forskali* and *H.* (*P.*) *sanctori* are basal compared to those containing only sulfated or mixed congeners (which are intermediate) [58]. On the generated phylogenetic tree established by [58], *H.* (*R.*) *arguinensis* was positioned in the same group as *H.* (*H.*) *tubulosa* and *H.* (*R.*) *poli* which present only sulfated saponins. The present study provides additional data by elucidating the saponins of *H.* (*R.*) *arguinensis* and *H.* (*H.*) *algeriensis* [which was not included in the tree generated by [58] as it was only recently described but according to [55], and [54], this species is genetically close to *H.* (*H.*) *tubulosa*] and shows that these two species do not exhibit only sulfated saponins but rather a mixture of both (Figure 6). However, since the characterization of the saponin profile of *H.* (*H.*) *tubulosa* and *H.* (*R.*) *poli* is not recent, further analyses should be conducted on their saponins and would provide answers on the presence of these species in the same group. On the other hand, the elucidation of the complete saponins profile of *H.* (*H.*) *tubulosa* should allow us to confirm the status of *H.* (*H.*) *algeriensis* using chemotaxonomy, and its separation following morphological [54] and phylogenetic studies [54,55].

The high diversity of saponins in sea cucumbers is related to their broad ecological functions including chemical defense and intra and inter-specific chemical communication [17,32,34,37,38,58,62]. Considering, for example, chemical defense, the diversity and concentration of triterpene glycosides in sea cucumbers appears to be correlated with the fact that they possess defensive organs (Cuvierian tubules) or not [16,17,63]. In fact, among the Holothuriidae, the genus *Bohadschia*, which has well-developed Cuvierian tubules with expellability and stickiness, has non-sulfated and less oxidized glycosides in both Cuvierian tubules and the body wall [22,63,64]. This is also the case of *H.* (*P.*) *sanctori* and *H.* (*P.*) *forskali* which possess Cuvierian tubules and present only non-sulfated saponins [17,42]. However, more sulfated and oxidized glycosides have been reported in species without or with dysfunctional Cuvierian tubules [63], as these saponins [61] are considered more toxic/repellent [65].

Within their broad ecological functions, triterpene glycosides exhibit different biological activities that could contribute to the probability of survival of producing organisms [22]. Depending on the taxonomic group of sea cucumbers, the number, composition, and location of monosaccharide units as well as the position of functional groups in the holostane backbone (i.e., hydroxyl, acetyl, sulfate, double bonds, etc.) may affect the bioactivity of the compounds [62,66]. Driving forces for evolution include optimization of biosynthesis, decreased metabolic cost, increased membranolytic action (toxicity), etc. [67]. Indeed, the absence of Cuvierian tubules which is compensated by the presence of sulfated saponins in more derived species may be explained by the biological function of the sulfate group that makes the saponin more toxic. It has been shown that the sulfate group of saponins plays a crucial role in the membranolytic activity of *Holothuria* (*Holothuria*) *scabra* (Jaeger, 1833) saponins and that desulfated saponins remain less active even at very high concentrations [68].

## 4. Materials and Methods

### 4.1. Chemicals

All technical grade methanol, hexane, chloroform, dichloromethane, isobutanol, HPLC grade water, and HPLC grade acetonitrile were provided by CHEM-LAB NV (Somme-Leuze, Belgium). The 2,5-dihydroxybenzoic acid (DHB) and *N*,*N*-dimethylaniline (DMA) were purchased from Sigma-Aldrich (Diegem, Belgium). VWR Chemicals (Leuven, Belgium) provided sodium citrate, sodium chloride, disodium phosphate dihydrate, potassium chloride, monopotassium phosphate, and borax.

### 4.2. Sampling

**Figure 7:**
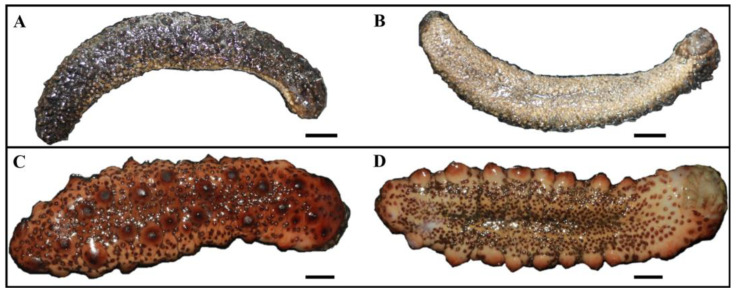
External morphology of *Holothuria* (*H.*) *algeriensis* (**A**) Dorsal face, (**B**) Ventral face and *Holothuria* (*R.*) *arguinensis* (**C**) Dorsal face, (**D**) Ventral face. Scale bars = 1 cm.

A total of three individuals of *Holothuria* (*H.*) *algeriensis* (Figure 7A,B) and two individuals of *Holothuria* (*R.*) *arguinensis* (Figure 7C,D) were studied. The specimens of *H.* (*R.*) *arguinensis* were collected in January 2020 and those of *H.* (*H.*) *algeriensis* in March 2021 by snorkeling in the region of Stidia (Mostaganem, Algeria) (35°50.061′ N, 0°00.830′ W) at 4 m depth. Each individual was measured contracted, weighed and then frozen at −80 °C in the case of *H.* (*R.*) *arguinensis* before being freeze-dried with the Christ Alpha 2-4 freeze-drier (Sigma, Germany). Those of *H.* (*H.*) *algeriensis* was frozen at −20 °C before being freeze-dried with the Christ Alpha 1-2 freeze-drier (Sigma, Germany). The whole body of the samples were then weighed, kept dry and then ground into powder for saponins extraction using Fritsch Pulverisette 2 grinder. The sex of individuals of both species was not determined since the individuals sampled were eviscerated because of stress.

The taxonomic identification of the specimens was carried out by the first and the second author, however, no specimens were deposited since the entire integument was used for saponin extraction.

### 4.3. Saponin Extraction

Extraction of saponins was performed following the protocol of [42] modified by [43]. The ground samples were placed overnight in methanol then filtered on Whatman filter paper 41 and 1. The filtrates were diluted with Milli-Q water to obtain a methanol concentration of 70%. Three liquid/liquid extractions followed, against *n*-hexane, dichloromethane, and chloroform, respectively. The recovered hydromethanolic fraction was evaporated at 46 °C using a rotary evaporator (IKA RV 10, IKA, Staufen, Germany) and the dry extract was recovered in a Milli-Q water/Iso-butanol (*v*/*v*) mixture. The butanolic fraction was rinsed once more with Milli-Q water and then its volume was reduced using a rotary evaporator to about 5 mL. The extract was recovered in previously weighed Eppendorf tubes and then dried completely using a Speed Vac (Jouan RC 10.22) by VWR Chemicals (Leuven, Belgium). The saponin concentration was calculated as the ratio of the dry weight of the saponin extract to the dry weight of the individuals. This value was not calculated for the species *H.* (*R.*) *arguinensis* since the weight of dry individuals was not taken.

### 4.4. Mass Spectrometry Analysis

Dry extracts of saponins from the two species were analyzed at the University of Mons (Organic Synthesis and Mass Spectrometry Lab) according to a well-established protocol based on mass spectrometry that combines MALDI-ToF, MALDI-HRMS, LC-MS, and LC-MS/MS analyses [43,68,69].

#### 4.4.1. MALDI-MS Analysis

For a general description of the saponin mixtures, the extracts were first analyzed by MALDI-ToF with the Waters Q-ToF Premier (Waters, Manchester, UK). The dry extracts were dissolved in a MilliQ water/acetonitrile solution (60/40) to reach a concentration of 1 mg/mL. The matrix was prepared from a mixture of 25 mg of 2,5-dihydroxybenzoic acid (DHB) and 6 µL of n,n-dimethylaniline (DMA) in 250 µL of Milli-Q water/acetonitrile (*v*/*v*). PEG600-2000 at a concentration of 1 mg/mL with a matrix composed of 4 mg of trans-2-[3-(4-tert-butylphenyl)-2-methyl-2-propenylidene] malononitrile (DCTB) diluted in 100 µL of chloroform was used to calibrate the mass spectrometer. The dry droplet method which consists of depositing 1 µL of samples on a drop of dry matrix was used.

The samples were analyzed in positive ion mode and the detected ions correspond to Na^+^ adducts of saponins and are therefore detected at an *m*/*z* = [M+23]. The MALDI source consists of a Nd-YAG laser, operating at 300 nm with a maximum pulse energy of 104.1 μJ delivered in 2.2 ns to the sample at a repetition rate of 200 Hz. For recording single-step MALDI-MS spectra, the quadrupole was set to pass ions between *m*/*z* 250 and 2000, and all ions were transmitted to the pusher region of the time-of-flight analyzer where they were mass analyzed with an integration time of 1 s. The mass analyses were performed with the ToF analyzer in reflectron mode, providing a resolution close to 10,000. The elemental composition of the detected ions was determined on the basis of accurate mass measurements (MALDI-HRMS) with PEG (polyethylene glycol) as external standard (lock mass).

For the quantitative analysis, we calculated the Isomer Molar Proportion (IMP, %) to represent the relative abundance of each saponin ion within the MALDI-MS mass spectrum. The ion counts for all isotopic signals corresponding to each saponin were summed, and the IMP was then determined by dividing this sum by the total ion counts for all detected saponins. For the saponin isomer relative quantification, the MALDI-MS quantitative data are crossed with the LC-MS data (see below) with the relative proportion of the different isomers being determined based on LC-MS signal integration. While we acknowledge that this method may not fully capture the precise saponin distribution within the extract, it provides a basis for direct comparison between different extracts.

#### 4.4.2. LC-MS Analysis

To investigate the possible presence of isomers, the extracts diluted to 0.1 mg/mL were analyzed by liquid chromatography-mass spectrometry (LC-MS) using the Waters Acquity UPLC H-Class (Waters, Manchester, UK) coupled to the Waters Synapt G2-S*i* mass spectrometer. This technique separates compounds according to their affinity with a non-polar column (Acquity UPLC BEH C18; 2.1 × 50 mm; 1.7 m, Waters, Manchester, UK) at 40 °C. A volume of 5 µL was injected on the column. The mobile phase was programmed with a constant flow rate of 250 µL/min and consisting of an elution gradient that starts with 85% of eluent A (Milli-Q water with 0.1% formic acid) and 15% of eluent B (Acetonitrile), reaches 60% of eluent A and 40% of eluent B at 6 min where it is maintained for 3 min. At 11 min, the ratio reaches 5% of eluent A and 95% of eluent B where it is maintained for 1 min then reduced to 85% of eluent A and 15% of eluent B at 13 min and maintained until the end of the chromatography (15 min).

Saponins ionization was produced by means of Electrospray Ionization (ESI) in positive mode generating [M+H]^+^ ions. The analyses were performed under the following conditions: source temperature 100 °C, desolvation temperature 300 °C, capillary voltage 3.1 kV, cone voltage 40 kV, source offset 80 V for a mass range between *m*/*z* 50 and 2000, with an integration time of 1s. Dry nitrogen was used as ESI gas with a flow rate of 500 L/h.

#### 4.4.3. LC-MS/MS Analysis

Tandem mass spectrometry coupled to liquid chromatography (LC-MS/MS) analysis were performed for structural characterization of the saponin ions previously identified by MALDI-MS and LC-MS. Ions of interest were mass selected by the quadrupole and subjected to collision-induced dissociation against argon (Ar) in the Trap cell of the Tri-Wave device and the kinetic energy of the laboratory frame (Elab) was set at 25 eV. The fragment ions were measured in mass with the ToF analyzer.

The identification of the saponins was then performed by comparing the MS/MS signature to the reported data for saponins identified in other species of the same family described by [16,17,37,42,57,70]. This last work represents a review of all saponin analyses performed on species of the genus *Holothuria*.

## 5. Conclusions

In this study, the saponin profiles of two sea cucumber species *H.* (*H.*) *algeriensis* and *H.* (*R.*) *arguinensis* are described, for the first time, using mass spectrometry methods, including MALDI-HRMS, LC-MS, and LC-MS/MS analyses. Eleven elemental compositions were detected in *H.* (*H.*) *algeriensis* and 18 in *H.* (*R.*) *arguinensis*. The results of LC-MS/MS allowed us to propose 13 new saponin molecules, six of which are specific to *H.* (*H.*) *algeriensis*, namely Saponins B, C, D, I, J, and K; three to *H.* (*R.*) *arguinensis*, namely Saponins 13, 14, 16; four are common to both species, namely Saponins A, E, F and G corresponding to Saponin 4, 6, 8, and 9, respectively. By comparing the saponins of the species studied with those of other species of the same genus and region, we found that they share non-sulfated saponins with *H.* (*P.*) *sanctori* and *H.* (*P.*) *forskali* and sulfated saponins with *H.* (*H.*) *tubulosa* and *H.* (*R.*) *poli*. However, the profile of *H.* (*H.*) *tubulosa* and *H.* (*R.*) *poli* is not well studied and it would be interesting to update the saponins analyses of these species with similar MS analyses. The results of this study are preliminary and require additional investigations, including NMR analyses for the chemical structures to be fully validated. However, they constitute a database for future studies that might focus on these species from the Algerian coast as they are increasingly overfished, consumed by the local population and illegally exported abroad. Determining their saponin profile and elucidating their biological activities could increase their commercial and pharmaceutical potential.

## Figures and Tables

**Table 1:** Mass spectrometry analysis of the saponin extract from *Holothuria* (*H.*) *algeriensis*. Elemental compositions and mass error (Δ) were determined by MALDI-High Resolution MS (MALDI-HRMS). Saponin names were assigned according to tandem MS (LC-MS/MS) results and comparisons with literature. The bolded names in the saponin column indicate the new saponins identified in the present study and the bolded numbers in the IMP (%) column indicate the isomers with the highest proportion for a given elemental composition. Underlined saponins are mentioned and explained in Section 3.1. ^a^ *m*/*z* value recorded with external calibration and *m*/*z* errors determined based on MALDI-HRMS measurements, ^b^ theoretical *m*/*z* values, ^c^ IMP (%) = Isomer Molar Proportions determined by signal integration (see experimental section), ^d^ due to the presence of the acidic-SO_3_H, the sulfated saponins appear as [M-H+2Na]^+^ ions upon MALDI-MS.

*m*/*z* ^a^ (Δ ppm) [M+Na]^+^	Saponin	*m*/*z_th_* ^b^ [M+Na]^+^	Elemental Composition (M)	Retention Time (min)	IMP (%) Individual 1 ^c^	IMP (%) Individual 2 ^c^	IMP (%) Individual 3 ^c^
1479.67 (1.1)	Holothurinoside I	1479.6620	C_67_H_108_O_34_	7.4	0.31	0.02	0.04
1463.66 (1.4)	Saponin A	1463.6671	C_67_H_108_O_33_	7.3	0.49	0.03	0.04
	Holothurinoside H/Marmoratoside B/17a-hydroxyimpatienside A			7.6	0.59	0.05	0.10
1461.67 (4.9)	Saponin B	1461.6725	C_64_H_110_O_35_	7.3	1.77	0.02	0.03
1449.66 (0)	Holothurinoside G	1449.6514	C_66_H_106_O_33_	7.1	0.36	0.02	0.05
1447.61 (4.9)	Saponin C	1447.6569	C_63_H_108_O_35_	7.1	1.36	0.01	0.03
	Saponin D			7.6	1.61	0.09	0.06
1287.59 (3.7)	Holothurinoside E	1287.5986	C_60_H_96_O_28_	7.1	0.07	0.11	0.08
	Holothurinoside O			7.6	0.20	0.35	0.26
1243.48 (2.1)	Scabraside B/25-hydroxyfuscocineroside B/Holothurin A	1243.4795 [M-H+2Na]^+ d^	C_54_H_86_O_27_S	7.6	44.51	49.36	37.20
	Saponin E			8.7	0.35	0.05	0.24
1227.52 (3.4)	Scabraside A/24-dehydroechinoside A/Fuscocineroside C/Fuscocineroside B	1227.4845 [M-H+2Na]^+ d^	C_54_H_86_O_26_S	7.6	0.37	0.29	0.27
	Saponin F			7.9	2.29	3.45	4.76
	Saponin G			11.3	0.31	0.15	0.39
1225.56 (2.2)	Saponin H	1225.5689 [M-H+2Na]^+ d^	C_54_H_84_O_26_S	7.6	1.45	1.04	1.23
	Saponin I			7.9	0.42	0.54	1.78
1111.44 (2.6)	Saponin J	1111.5665	C_54_H_88_O_22_	9.3	0.06	0.08	0.97
	Saponin K			7.6	0.08	0.04	0.08
905.41 (2.8)	Holothurin B/Holothurin B4	905.3582 [M-H+2Na]^+ d^	C_41_H_64_O_17_S	7.6	33.21	31.98	28.04
	Nobiliside B (I)			8.5	0.06	0.16	1.86
	Holothurin B/Holothurin B4			10.1	11.48	12.18	22.55

**Table 2:** Mass spectrometry analysis of the saponin extract from *Holothuria* (*R.*) *arguinensis*. Elemental compositions and mass error (Δ) measurements were determined by MALDI-HRMS. IMP (%) = Isomer Molar Proportion. Saponin names were assigned according to LC-MS/MS results. The bolded names in the saponin column indicate the new saponins identified in the present study and the bolded numbers in the IMP (%) column indicate the isomers with highest proportions for a given elemental composition. Underlined saponins are mentioned and explained in Section 3.1. ^a^ *m*/*z* value recorded with external calibration and *m*/*z* errors determined based on MALDI-HRMS measurements, ^b^ theoretical *m*/*z* values, ^c^ IMP (%) = Isomer Molar Proportions determined by signal integration (see experimental section), ^d^ due to the presence of the acidic -SO_3_H, the sulfated saponins appear as [M-H+2Na]^+^ ions upon MALDI-MS.

*m*/*z* ^a^ (Δ ppm) [M+Na]^+^	Saponin	*m*/*z* _th_ ^b^ [M+Na]^+^	Elemental Composition (M)	Retention Time (min)	IMP (%) Individual 1 ^c^	IMP (%) Individual 2 ^c^
1509.74 (0.1)	Saponin 1	1509.6725	C_68_H_110_O_35_	7.6	0.13	0.12
	Saponin 2			8.2	0.02	0.03
1495.74 (3.1)	Saponin 3	1495.6569	C_67_H_108_O_35_	8.2	0.04	0.18
1463.71 (2.2)	Holothurinoside H/Marmoratoside B/ 17a-hydroxyimpatienside A	1463.6671	C_67_H_108_O_33_	7.1	0.48	0.12
	Saponin 4			7.3	0.13	0.10
	Saponin 5			8.4	0.11	0.49
1355.67 (0.1)	Unidentified	1355.5835 [M-H+2Na]^+ d^	C_65_H_98_O_25_S	7.6	0.02	0.03
1317.61 (1.1)	Holothurinoside N	1317.6091	C_61_H_98_O_29_	7.6	0.03	0.02
1261.50 (4.1)	Unidentified	1261.4900 [M-H+2Na]^+ d^	C_54_H_88_O_28_S	7.6	0.57	0.45
1243.50 (0.2)	Saponin 6	1243.4795 [M-H+2Na]^+ d^	C_54_H_86_O_27_S	7.4	0.86	0.62
	Scabraside B/25-hydroxyfuscocineroside B/Holothurin A			7.6	12.40	8.99
1229.52 (3.9)	Unidentified	1229.4637 [M-H+2Na]^+ d^	C_53_H_84_O_27_S	7.4	0.16	0.03
	Saponin 7			11.3	0.87	1.04
1227.52 (1.1)	Saponin 8	1227.4845 [M-H+2Na]^+ d^	C_54_H_86_O_26_S	7.6	0.09	0.08
	Scabraside A/24-dehydroechinoside A/Fuscocineroside C/Fuscocineroside B			7.9	0.93	1.25
	Saponin 9			11.3	0.55	0.50
1209.49 (4.2)	Saponin 10	1209.4740 [M-H+2Na]^+ d^	C_54_H_84_O_25_S	7.4	0.16	0.03
1171.57 (4.2)	Unidentified	1171.5513	C_55_H_88_O_25_	7.6	0.15	0.10
1157.59 (0.4)	Unidentified	1157.5356	C_54_H_86_O_25_	7.6	0.14	0.20
1141.59 (2.8)	Desholothurin A	1141.5406	C_54_H_86_O_24_	7.6	0.14	0.09
	Saponin 11			10.1	0.47	1.07
	Saponin 12			13.6	0.04	0.04
1125.58 (5.0)	Saponin 13	1125.5458	C_54_H_86_O_23_	7.6	0.41	0.57
	Holothurinoside C			10.1	0.97	3.72
995.44 (1.2)	Saponin 14	995.4828	C_48_H_76_O_20_	10.1	0.01	0.05
951.57 (4.8)	Saponin 15	951.4929	C_47_H_76_O_18_	11.6	0.22	0.08
905.41 (0.3)	Nobiliside B (I)	905.3582 [M-H+2Na]^+ d^	C_41_H_64_O_17_S	7.4	1.68	0.09
	Holothurin B/Holothurin B4			7.6	9.81	7.28
	Holothurin B/Holothurin B4			10.0	64.50	67.62
889.43 (0.1)	Holothurin B3/24-dehydroechinoside B	889.3632 [M-H+2Na]^+ d^	C_41_H_64_O_16_S	7.9	0.77	1.09
	Holothurin B3/24-dehydroechinoside B			10.9	2.84	3.77
	Saponin 16			12.0	0.32	0.16

**Table 3:** Overview of saponins identified in *Holothuria* (*H.*) *algeriensis* and *Holothuria* (*R.*) *arguinensis* and their structural properties (X = present). Letters and numbers in the Aglycone-R1, R2, and R3 columns correspond to the substructures shown in Figure 3, Figure 4, and Figure 5, respectively. In the “Saponin” column, bolded name indicates new structures identified in this study and, in the “Aglycone-R1” column, bolded information indicates the most likely structure.

Saponin	Molecular Formula	Aglycone-R1	R2	R3	*m*/*z* [M+Na]^+^	Nominal Mass	*H.* (*H.*) *algeriensis*	*H.* (*R.*) *arguinensis*
**Saponin 1**	C_68_H_110_O_35_	B11/F2	g	VIII	1509	1486		X
**Saponin 2**		Unidentified	g	IX				X
**Saponin 3**	C_67_H_108_O_35_	Unidentified	g	VIII	1495	1472		X
Holothurinoside I	C_67_H_108_O_34_	B1	f	VI	1479	1456	X	
17a-hydroxyimpatienside A	C_67_H_108_O_33_	B4	f	VI	1463	1440	**X**	**X**
Holothurinoside H		A1	f	VI			**X**	**X**
Marmoratoside B		A10	f	VI			**X**	**X**
**Saponin 5**		Unidentified	k	VII				X
**Saponin A (=Saponin 4)**		A9/**B1**/B8/B10	f	IX			X	X
Holothurinoside G	C_66_H_106_O_33_	B1	f	VIII	1449	1426	X	
**Saponin B**	C_64_H_110_O_35_	Unidentified	f	VI	1461	1438	X	
**Saponin C**	C_63_H_108_O_35_	A9/**B1**/B8/B10	f	X	1447	1424	X	
**Saponin D**		**A1**/A8/A10/B4/B5/D1	f	XI			X	
Holothurinoside N (=Holothurinoside L)	C_61_H_98_O_29_	B1	f	IV	1317	1294		X
Holothurinoside E	C_60_H_96_O_28_	A1	f	III	1287	1264	X	
Holothurinoside O		Unidentified	f	III			X	
**Saponin J**	C_54_H_88_O_22_	A4/A5/**C1**	e	I	1111	1088	X	
**Saponin K**		A9/**B1**/B8/B10	i	I			X	
25-hydroxyfuscocineroside B	C_54_H_86_O_27_S	A9	f	II	1243	1198	**X**	**X**
Holothurin A		B1	f	II			**X**	**X**
**Saponin E (=Saponin 6)**		**A1**/A8/A10/B4/B5/D1	g	II			**X**	**X**
Scabraside B (=17-hydroxyfuscocineroside B)		B8	f	II			**X**	**X**
17-dehydroxyholothurin A (=Fuscocineroside C)	C_54_H_86_O_26_S	A1	f	II	1227	1182	**X**	**X**
24-Dehydroechinoside A		B4	f	II			**X**	**X**
Fuscocineroside B		A8	f	II			**X**	**X**
**Saponin F (=Saponin 8)**		A9/**B1**/B8/B10	h	II			**X**	**X**
**Saponin G (=Saponin 9)**		A4/A5/**C1**	g	II			**X**	**X**
Scabraside A		B5	f	II			**X**	**X**
**Saponin 7**	C_53_H_84_O_27_S	A6/B2	f	II				X
**Saponin 10**	C_54_H_84_O_25_S	Unidentified	h	II	1209	1164		X
Desholothurin A (=Nobiliside 2A)	C_54_H_86_O_24_	B1	f	I	1141	1118		X
**Saponin 11**		B11/F2	j	I				X
**Saponin 12**		Unidentified	i	I				X
Holothurinoside C	C_54_H_86_O_23_	A1	f	I	1125	1102		X
**Saponin 13**		A9/**B1**/B8/B10	h	I				X
**Saponin H**	C_54_H_84_O_26_S	A12	d	II	1225	1180	X	
**Saponin I**		E4	j	II			X	
**Saponin 14**	C_48_H_76_O_20_	F7	b	V	995	972		X
**Saponin 15**	C_47_H_76_O_18_	A6/B2	c	IV	951	928		X
Holothurin B	C_41_H_64_O_17_S	B1	b	II	905	860	**X**	**X**
Holothurin B4		B10	b	II			**X**	**X**
**Nobiliside B (I)** **[=Nobiliside C (II)]**		D1	a	II			**X**	**X**
24-dehydroechinoside B	C_41_H_64_O_16_S	B4	b	II	889	844		X
Holothurin B3		A1	b	II				X
**Saponin 16**		A4/A5/**C1**	a	II				X

**Table 4:** Saponins shared between *Holothuria* species occurring on the Algerian coast (X = present). Saponins in bold are different possibilities reported for *H. (H.) algeriensis* and *H. (R.) arguinensis* for the same molecular formula (aglycone not confirmed).

Saponin	Molecular Formula	*H.* (*H.*) *algeriensis*	*H.* (*R.*) *arguinensis*	*H.* (*R.*) *poli*	*H.* (*H.*) *tubulosa*	*H.* (*P.*) *sanctori*	*H.* (*P.*) *forskali*
Holothurinoside I	C_67_H_108_O_34_	X					X
**17a-hydroxyimpatienside A**	C_67_H_108_O_33_	X	X				
**Holothurinoside H**		X	X				X
**Marmoratoside B**		X	X				X
Saponine A (=Saponine 4)		X	X				
Holothurinoside G	C_66_H_106_O_33_	X					X
Holothurinoside N (=Holothurinoside L)	C_61_H_98_O_29_		X			X	
Holothurinoside E	C_60_H_96_O_28_	X				X	X
**25-hydroxyfuscocineroside B**	C_54_H_86_O_27_S	X	X				
**Holothurin A**		X	X	X	X		
Saponine E (=Saponine 6)		X	X				
**Scabraside B (=17-hydroxyfuscocineroside B)**		X	X				
**17-dehydroxyholothurin A (=Fuscocineroside C)**	C_54_H_86_O_26_S	X	X				
**24-Dehydroechinoside A**		X	X				
**Fuscocineroside B**		X	X				
Saponine F (=Saponine 8)		X	X				
Saponine G (=Saponine 9)		X	X				
**Scabraside A**		X	X				
Desholothurin A (=Nobiliside 2A)	C_54_H_86_O_24_		X				X
Holothurinoside C	C_54_H_86_O_23_		X			X	X
**Holothurin B**	C_41_H_64_O_17_S	X	X	X	X		
**Holothurin B4**		X	X	X			
Holothurin B3	C_41_H_64_O_16_S		X	X			

## Data Availability

The data presented in this study are available on request from the corresponding author.

## Supplementary Materials

The following supporting information can be downloaded at: https://www.mdpi.com/article/10.3390/molecules29225346/s1. Figure S1. (A) Matrix-assisted Laser Desorption/Ionization mass spectrum [MALDI-MS (+)] of the total saponin mixture of the second individual of *Holothuria* (*H.*) *algeriensis* integument extracts. (B) MALDI-MS (+) mass spectrum of the total saponin mixture of the third individual of *Holothuria* (*H.*) *algeriensis* integument extracts. Saponins signals are highlighted by black dots on which the [M+Na]^+^ values are noted. Figure S2. Matrix-assisted Laser Desorption/Ionization mass spectrum [MALDI-MS (+)] of the total saponin mixture of the second individual of *Holothuria* (*R.*) *arguinensis* integument extracts. Saponin signals are highlighted by black dots on which the *m*/*z* values for [M+Na]^+^ ions are noted. Figure S3. (A) MALDI-HRMS(+) mass spectrum of the first individual of *Holothuria* (*H.*) *algeriensis*. (B) MALDI-HRMS(+) mass spectrum of the second individual of *Holothuria* (*H.*) *algeriensis*. (C) MALDI-HRMS(+) mass spectrum of the third individual of *Holothuria* (*H.*) *algeriensis*. Note that PEG (polyethylene glycol) was used as the internal reference (lock mass). Figure S4. (A) MALDI-HRMS(+) mass spectrum of the first individual of *Holothuria* (*R.*) *arguinensis*. (B) MALDI-HRMS(+) mass spectrum of the second individual of *Holothuria* (*R.*) *arguinensis*. Note that PEG (polyethylene glycol) was used as the standard reference. Figure S5. LC-MS analysis of the *Holothuria* (*H.*) *algeriensis* saponin extract: EIC (Extracted Ion Current Chromatogram) of the *m*/*z* 1425 [M+H]^+^ ions. Figure S6. LC-MS analysis of a *Holothuria* (*H.*) *algeriensis* saponin extract: mass spectrum at 7.6 min retention time. Figure S7. LC-MS/MS(+) analysis of *H.* (*H.*) *algeriensis* saponin extract: CID spectrum recorded for the *m*/*z* 1439 precursor ions [M+H]^+^ at 7.3 min retention time (Saponin B). Figure S8. LC-MS/MS(+) analysis of *H.* (*H.*) *algeriensis* saponin extract: CID spectrum recorded for the *m*/*z* 1425 precursor ions [M+H]^+^ at 7.1 min retention time (Saponin C). Figure S9. LC-MS/MS(+) analysis of *H.* (*H.*) *algeriensis* saponin extract: CID spectrum recorded for the *m*/*z* 1425 precursor ions [M+H]^+^ at 7.6 min retention time (Saponin D). Figure S10. LC-MS/MS(+) analysis of *H.* (*H.*) *algeriensis* saponin extract: CID spectrum recorded for the *m*/*z* 1181 precursor ions [M+H]^+^ at 8.7 min retention time (Saponin E). Figure S11. LC-MS/MS(+) analysis of *H.* (*H.*) *algeriensis* saponin extract: CID spectrum recorded for the *m*/*z* 1165 precursor ions [M+H]^+^ at 7.9 min retention time (Saponin F). Figure S12. LC-MS/MS(+) analysis of *H.* (*H.*) *algeriensis* saponin extract: CID spectrum recorded for the *m*/*z* 1165 precursor ions [M+H]^+^ at 11.3 min retention time (Saponin G). Figure S13. LC-MS/MS(+) analysis of *H.* (*H.*) *algeriensis* saponin extract: CID spectrum recorded for the *m*/*z* 1163 precursor ions [M+H]^+^ at 7.6 min retention time (Saponin H). Figure S14. LC-MS/MS(+) analysis of *H.* (*H.*) *algeriensis* saponin extract: CID spectrum recorded for the *m*/*z* 1163 precursor ions [M+H]^+^ at 7.9 min retention time (Saponin I). Figure S15. LC-MSMS(+) analysis of *H.* (*H.*) *algeriensis* saponin extract: CID spectrum recorded for the *m*/*z* 1089 precursor ions [M+H]^+^ at 9.3 min retention time (Saponin J). Figure S16. LC-MSMS(+) analysis of *H.* (*H.*) *algeriensis* saponin extract: CID spectrum recorded for the *m*/*z* 1089 precursor ions [M+H]^+^ at 7.6 min retention time (Saponin K). Figure S17. LC-MS/MS(+) analysis of *H.* (*R.*) *arguinensis* saponin extract: CID spectrum recorded for the *m*/*z* 1487 precursor ions [M+H]^+^ at 7.6 min retention time (Saponin 1). Figure S18. LC-MS/MS(+) analysis of *H.* (*R.*) *arguinensis* saponin extract: CID spectrum recorded for the *m*/*z* 1487 precursor ions [M+H]^+^ at 8.2 min retention time (Saponin 2). Figure S19. LC-MS/MS(+) analysis of *H.* (*R.*) *arguinensis* saponin extract: CID spectrum recorded for the *m*/*z* 1473 precursor ions [M+H]^+^ at 8.2 min retention time (Saponin 3). Figure S20. LC-MS/MS(+) analysis of *H.* (*R.*) *arguinensis* saponin extract: CID spectrum recorded for the *m*/*z* 1141 precursor ions [M+H]^+^ at 7.3 min retention time (Saponin 4). Figure S21. LC-MS/MS(+) analysis of *H.* (*R.*) *arguinensis* saponin extract: CID spectrum recorded for the *m*/*z* 1141 precursor ions [M+H]^+^ at 8.4 min retention time (Saponin 5). Figure S22. LC-MS/MS(+) analysis of *H.* (*R.*) *arguinensis* saponin extract: CID spectrum recorded for the *m*/*z* 1181 precursor ions [M+H]^+^ at 7.4 min retention time (Saponin 6). Figure S23. LC-MS/MS(+) analysis of *H.* (*R.*) *arguinensis* saponin extract: CID spectrum recorded for the *m*/*z* 1167 precursor ions [M+H]^+^ at 11.3 min retention time (Saponin 7). Figure S24. LC-MS/MS(+) analysis of *H.* (*R.*) *arguinensis* saponin extract: CID spectrum recorded for the *m*/*z* 1165 precursor ions [M+H]^+^ at 7.6 min retention time (Saponin 8). Figure S25. LC-MS/MS(+) analysis of *H.* (*R.*) *arguinensis* saponin extract: CID spectrum recorded for the *m*/*z* 1165 precursor ions [M+H]^+^ at 11.3 min retention time (Saponin 9). Figure S26. LC-MS/MS(+) analysis of *H.* (*R.*) *arguinensis* saponin extract: CID spectrum recorded for the *m*/*z* 1147 precursor ions [M+H]^+^ at 7.4 min retention time (Saponin 10). Figure S27. LC-MS/MS(+) analysis of *H.* (*R.*) *arguinensis* saponin extract: CID spectrum recorded for the *m*/*z* 1119 precursor ions [M+H]^+^ at 10.1 min retention time (Saponin 11). Figure S28. LC-MS/MS(+) analysis of *H.* (*R.*) *arguinensis* saponin extract: CID spectrum recorded for the *m*/*z* 1119 precursor ions [M+H]^+^ at 13.6 min retention time (Saponin 12). Figure S29. LC-MS/MS(+) analysis of *H.* (*R.*) *arguinensis* saponin extract: CID spectrum recorded for the *m*/*z* 1103 precursor ions [M+H]^+^ at 7.6 min retention time (Saponin 13). Figure S30. LC-MS/MS(+) analysis of *H.* (*R.*) *arguinensis* saponin extract: CID spectrum recorded for the *m*/*z* 973 precursor ions [M+H]^+^ at 10.1 min retention time (Saponin 14). Figure S31. LC-MS/MS(+) analysis of *H.* (*R.*) *arguinensis* saponin extract: CID spectrum recorded for the *m*/*z* 929 precursor ions [M+H]^+^ at 11.6 min retention time (Saponin 15). Figure S32. LC-MS/MS(+) analysis of *H.* (*R.*) *arguinensis* saponin extract: CID spectrum recorded for the *m*/*z* 827 precursor ions [M+H]^+^ at 12.0 min retention time (Saponin 16).
