# Supplementary material for: Structural Characterization and Profiles of Saponins from Two Algerian Sea Cucumbers
Source: Molecules. 2024 Nov 13;29(22):5346. doi: 10.3390/molecules29225346 (PMC11596277; doi:10.3390/molecules29225346)
Supplement: Supplementary file 1 [file molecules-29-05346-s001.zip › molecules-3190222-supplementary.pdf]

Structural Characterization and Profiles of Saponins from Two Algerian Sea Cucumbers

Ihcene Khodja [1], Karim Mezali [1,*], Philippe Savarino [2], Pascal Gerbaux [2,*], Patrick Flammang [3] and Guillaume Caulier [3]

[1] Protection, Valorization of Coastal Marine Resources and Molecular Systematics Laboratory, Department of Marine Science and Aquaculture, Faculty of Natural Sciences and Life, Abdelhamid Ibn Badis University—Mostaganem, Route nationale N° 11, P.O. Box 227, Kharrouba, Mostaganem 27000, Algeria; ihcene.khodja.etu@univ-mosta.dz

[2] Research Institute for Biosciences, Organic Synthesis and Mass Spectrometry Laboratory, University of Mons—UMONS, 23, Place du Parc, B-7000 Mons, Belgium; philippe.savarino@outlook.fr

[3] Research Institute for Biosciences, Biology of Marine Organisms and Biomimetics Unit, University of Mons—UMONS, 23, Place du Parc, B-7000 Mons, Belgium; patrick.flammang@umons.ac.be (P.F.); guillaume.caulier@umons.ac.be (G.C.)

* Correspondence: karim.mezali@univ-mosta.dz (K.M.); pascal.gerbaux@umons.ac.be (P.G.)

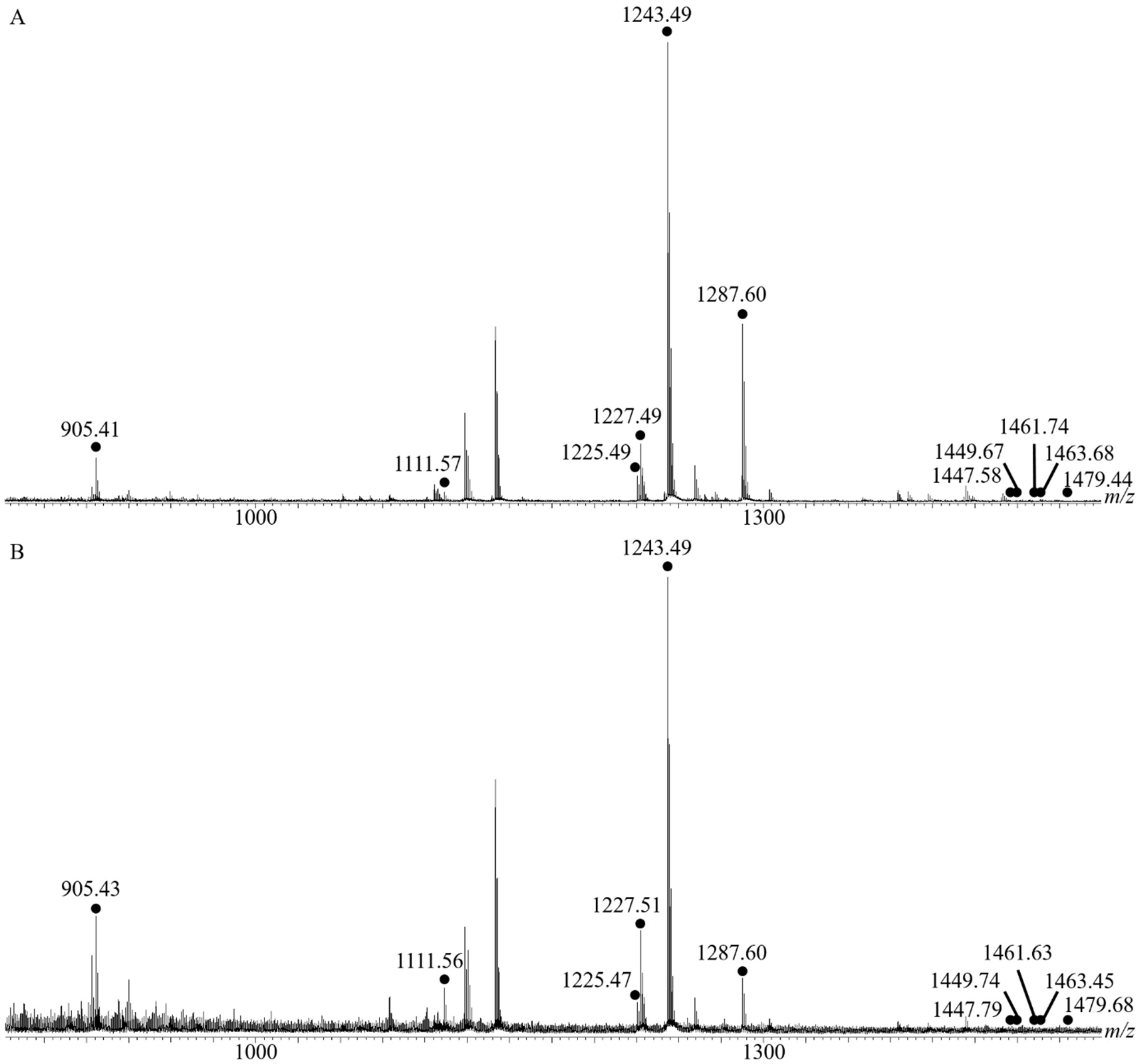

Figure S1. (A) Matrix-assisted Laser Desorption/Ionization mass spectrum [MALDI-MS (+)] of the total saponin mixture of the second individual of *Holothuria* (*H.*) *algeriensis* integument extracts. **(B)** MALDI-MS (+) mass spectrum of the total saponin mixture of the third individual of *Holothuria* (*H.*) *algeriensis* integument extracts. Saponins signals are highlighted by black dots on which the $[M+Na]^+$ values are noted.

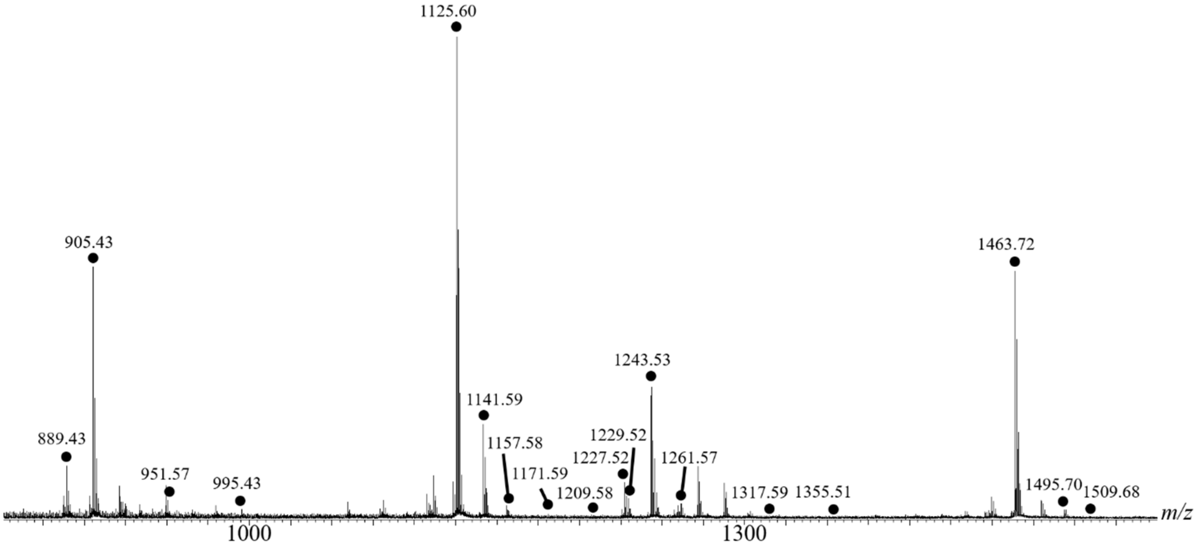

Figure S2. Matrix-assisted Laser Desorption/Ionization mass spectrum [MALDI-MS (+)] of the total saponin mixture of the second individual of *Holothuria* (*R.*) *arguinensis* integument extracts. Saponin signals are highlighted by black dots on which the *m/z* values for $[M+Na]^+$ ions are noted.

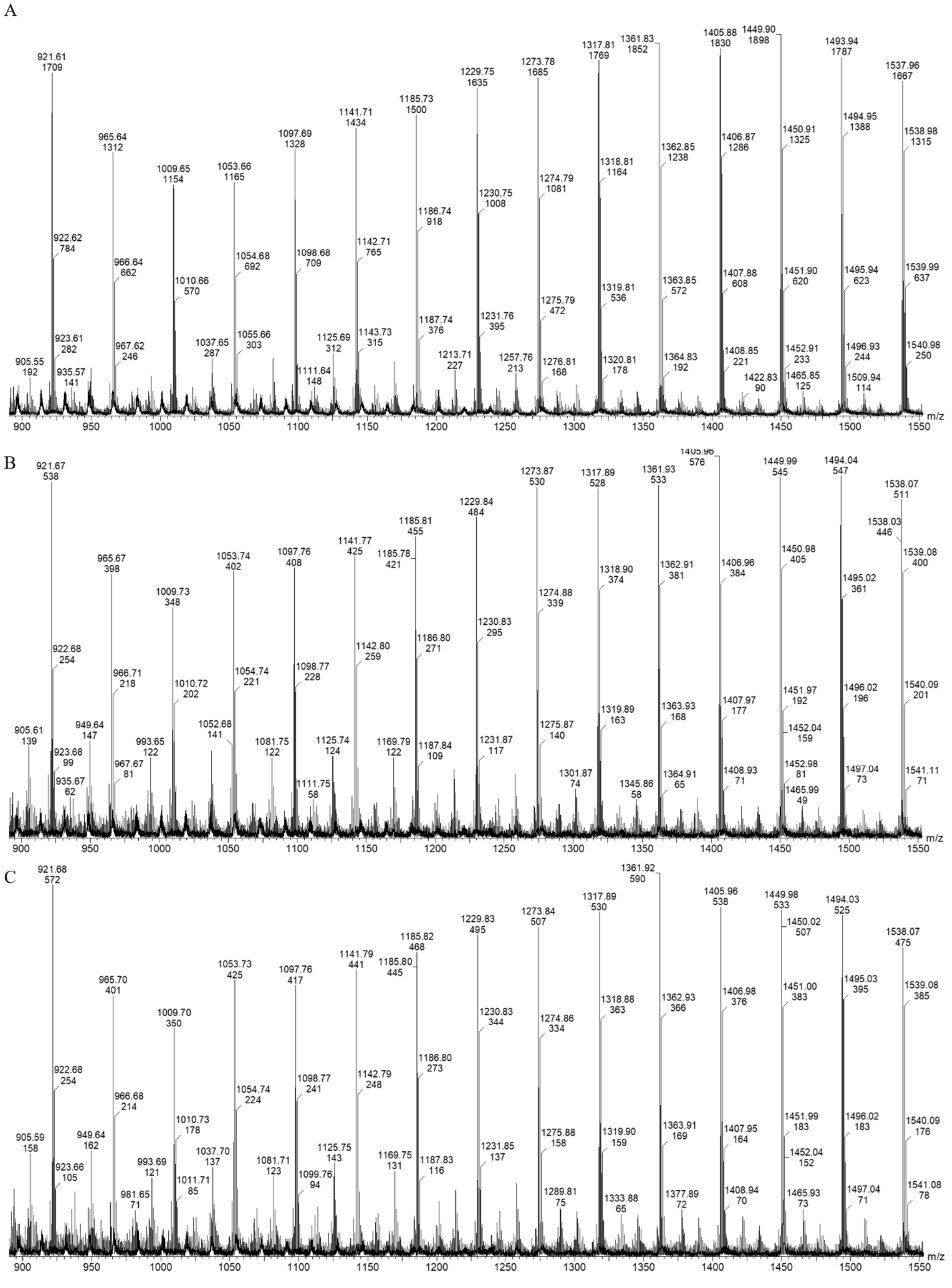
A
905.55 192
921.61 1709
922.62 784
923.61 282
935.57 141
965.64 1312
966.64 662
967.62 246
1009.65 1154
1010.66 570
1037.65 287
1053.66 1165
1054.68 692
1055.66 303
1097.69 1328
1098.68 709
1111.64 148
1125.69 312
1141.71 1434
1142.71 765
1143.73 315
1185.73 1500
1186.74 918
1187.74 376
1213.71 227
1229.75 1635
1230.75 1008
1231.76 395
1257.76 213
1273.78 1685
1274.79 1081
1275.79 472
1276.81 168
1317.81 1769
1318.81 1164
1319.81 536
1320.81 178
1361.83 1852
1362.85 1238
1363.85 572
1364.83 192
1405.88 1830
1406.87 1286
1407.88 608
1408.85 221
1422.83 90
1449.90 1898
1450.91 1325
1451.90 620
1452.91 233
1465.85 125
1493.94 1787
1494.95 1388
1495.94 623
1496.93 244
1509.94 114
1537.96 1667
1538.98 1315
1539.99 637
1540.98 250
m/z
900 950 1000 1050 1100 1150 1200 1250 1300 1350 1400 1450 1500 1550
B
905.61 139
921.67 538
922.68 254
923.68 99
935.67 62
949.64 147
965.67 398
966.71 218
967.67 81
993.65 122
1009.73 348
1010.72 202
1052.68 141
1053.74 402
1054.74 221
1081.75 122
1097.76 408
1098.77 228
1111.75 58
1125.74 124
1141.77 425
1142.80 259
1169.79 122
1185.78 421
1185.81 455
1186.80 271
1187.84 109
1229.84 484
1230.83 295
1231.87 117
1273.87 530
1274.88 339
1275.87 140
1301.87 74
1317.89 528
1318.90 374
1319.89 163
1345.86 58
1361.93 533
1362.91 381
1363.93 168
1364.91 65
1405.96 576
1406.96 384
1407.97 177
1408.93 71
1449.99 545
1450.98 405
1451.97 192
1452.04 159
1452.98 81
1465.99 49
1494.04 547
1495.02 361
1496.02 196
1497.04 73
1538.07 511
1538.03 446
1539.08 400
1540.09 201
1541.11 71
m/z
900 950 1000 1050 1100 1150 1200 1250 1300 1350 1400 1450 1500 1550
C
905.59 158
921.68 572
922.68 254
923.66 105
949.64 162
965.70 401
966.68 214
981.65 71
993.69 121
1009.70 350
1010.73 178
1011.71 85
1037.70 137
1053.73 425
1054.74 224
1081.71 123
1097.76 417
1098.77 241
1099.76 94
1125.75 143
1141.79 441
1142.79 248
1169.75 131
1185.80 445
1185.82 468
1186.80 273
1187.83 116
1229.83 495
1230.83 344
1231.85 137
1273.84 507
1274.86 334
1275.88 158
1289.81 75
1317.89 530
1318.88 363
1319.90 159
1333.88 65
1361.92 590
1362.93 366
1363.91 169
1377.89 72
1405.96 538
1406.98 376
1407.95 164
1408.94 70
1449.98 533
1450.02 507
1451.00 383
1451.99 183
1452.04 152
1465.93 73
1494.03 525
1495.03 395
1496.02 183
1497.04 71
1538.07 475
1539.08 385
1540.09 176
1541.08 78
m/z
900 950 1000 1050 1100 1150 1200 1250 1300 1350 1400 1450 1500 1550

Figure S3. (A) MALDI-HRMS(+) mass spectrum of the first individual of *Holothuria* (*H.*) *algeriensis*. **(B)** MALDI-HRMS(+) mass spectrum of the second individual of *Holothuria* (*H.*) *algeriensis*. **(C)** MALDI-HRMS(+) mass spectrum of the third individual of *Holothuria* (*H.*) *algeriensis***.** Note that PEG (polyethylene glycol) was used as the internal reference (lock mass).

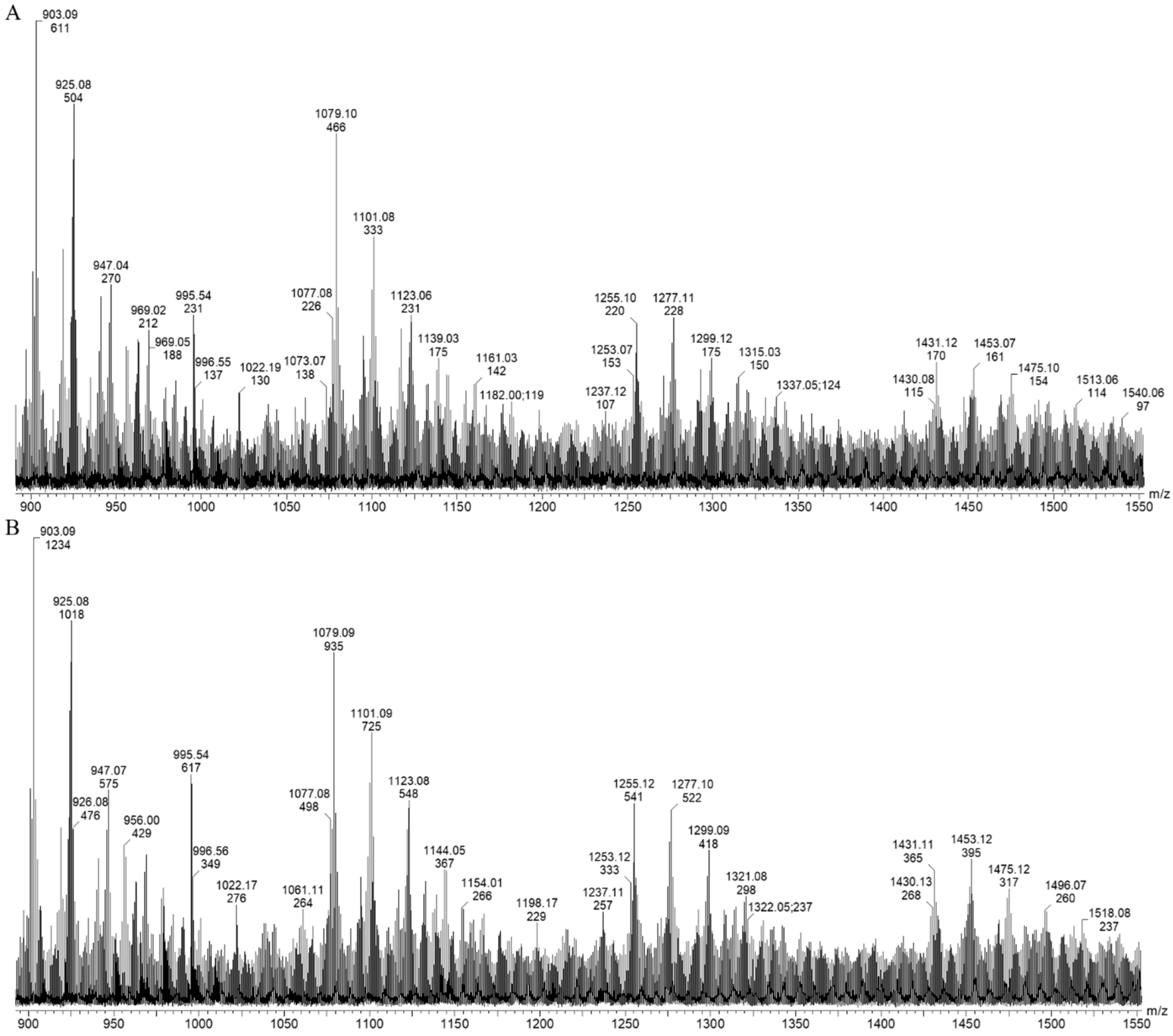

Figure S4. (A) MALDI-HRMS(+) mass spectrum of the first individual of *Holothuria* (*R.*) *arguinensis*. **(B)** MALDI-HRMS(+) mass spectrum of the second individual of *Holothuria* (*R.*) *arguinensis*. Note that PEG (polyethylene glycol) was used as the standard reference.

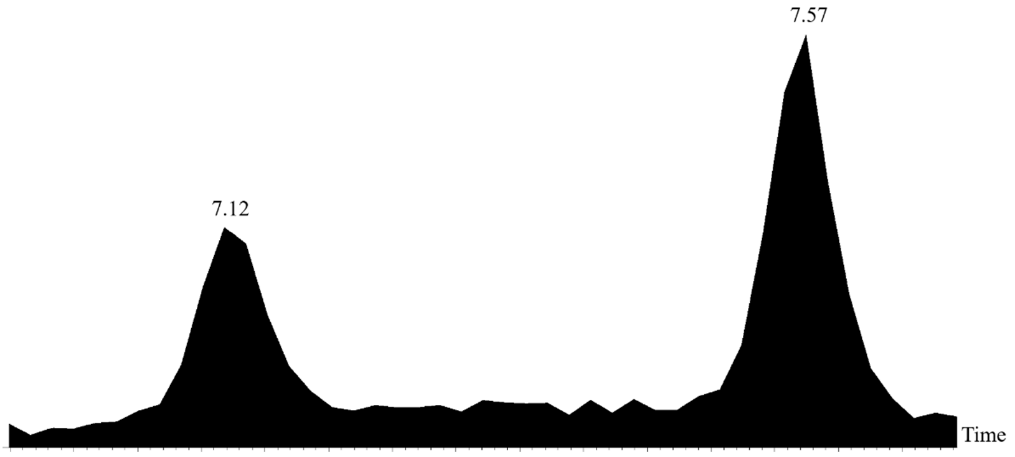

Figure S5. LC-MS analysis of the *Holothuria* (*H.*) *algeriensis* saponin extract: EIC (Extracted Ion Current Chromatogram) of the *m/z* 1425 $[M+H]^+$ ions.

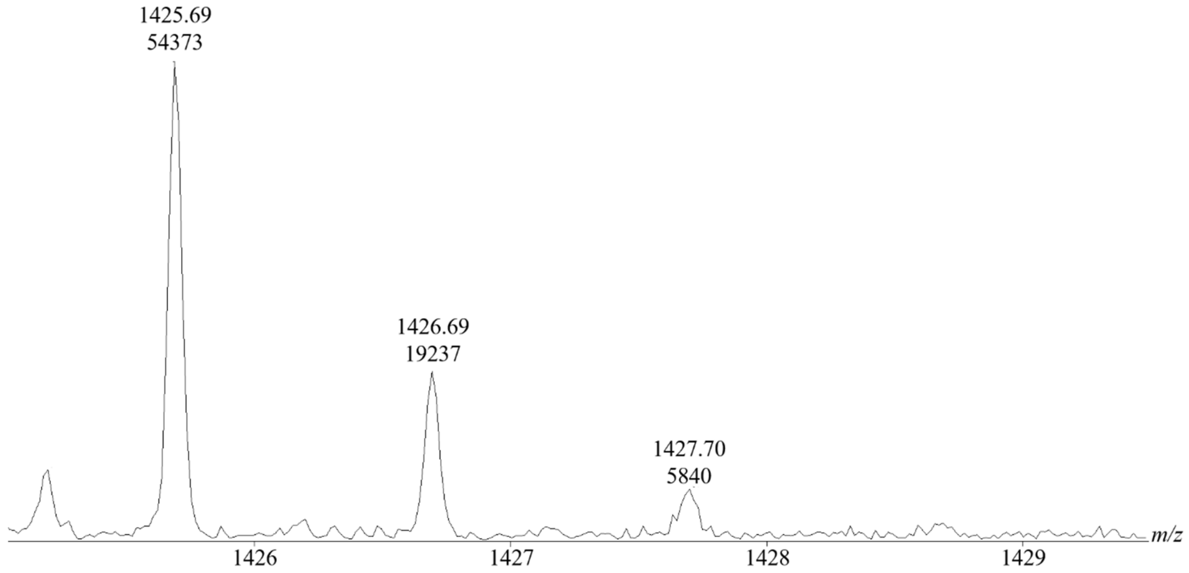

Figure S6. LC-MS analysis of a *Holothuria* (*H.*) *algeriensis* saponin extract : mass spectrum at 7.6 min retention time.

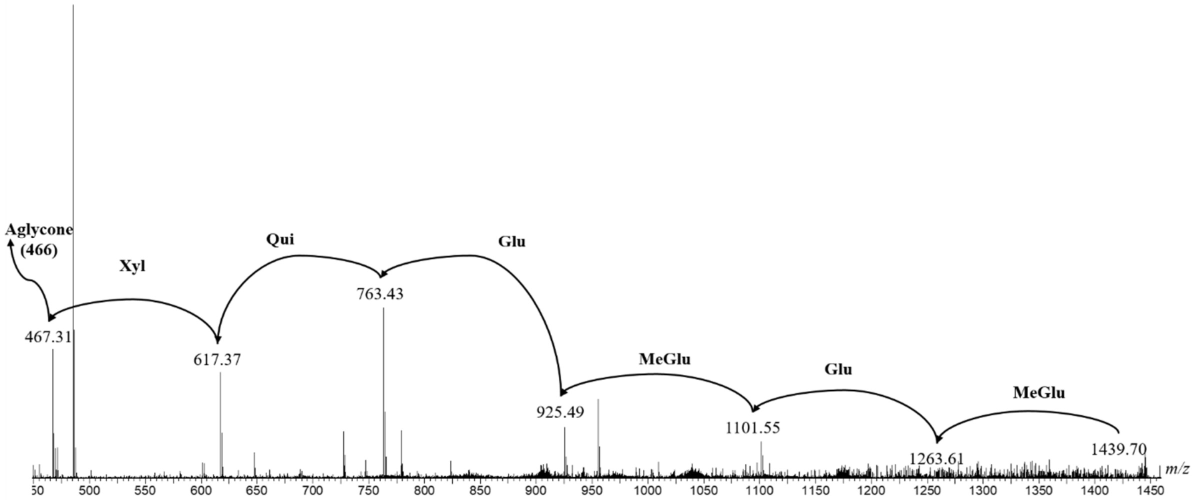

Figure S7. LC-MS/MS(+) analysis of *H.* (*H.*) *algeriensis* saponin extract: CID spectrum recorded for the m/z 1439 precursor ions [M+H]+ at 7.3 min retention time (Saponin B).

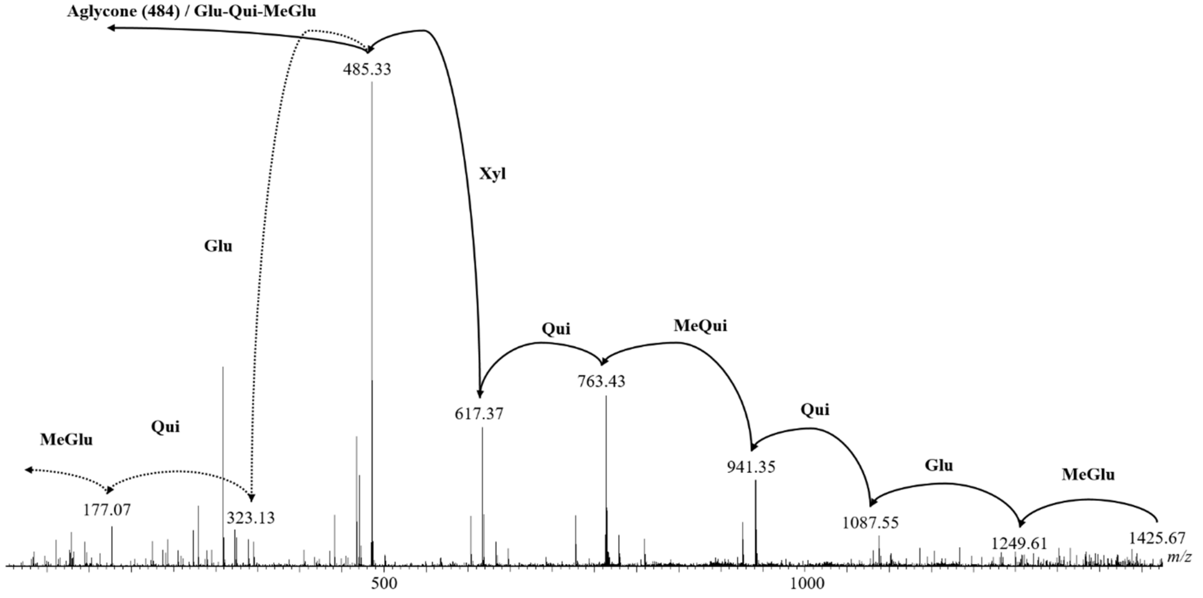

Figure S8. LC-MS/MS(+) analysis of *H.* (*H.*) *algeriensis* saponin extract: CID spectrum recorded for the *m/z* 1425 precursor ions [M+H]+ at 7.1 min retention time (Saponin C).

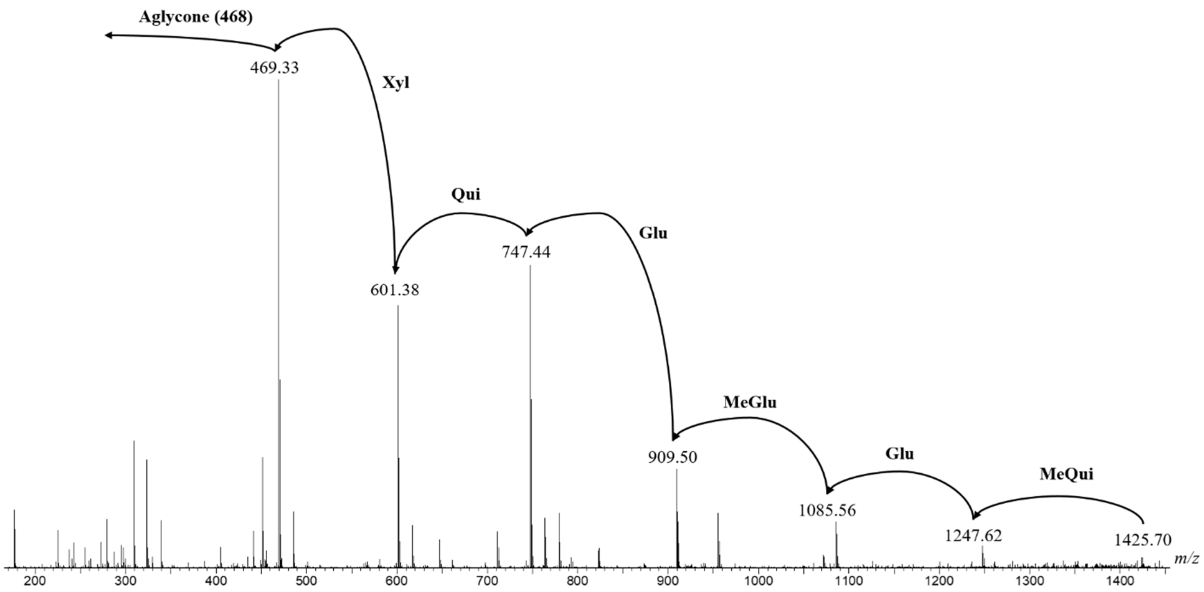

Figure S9. LC-MS/MS(+) analysis of *H.* (*H.*) *algeriensis* saponin extract: CID spectrum recorded for the *m*/*z* 1425 precursor ions $[M+H]^+$ at 7.6 min retention time (Saponin D).

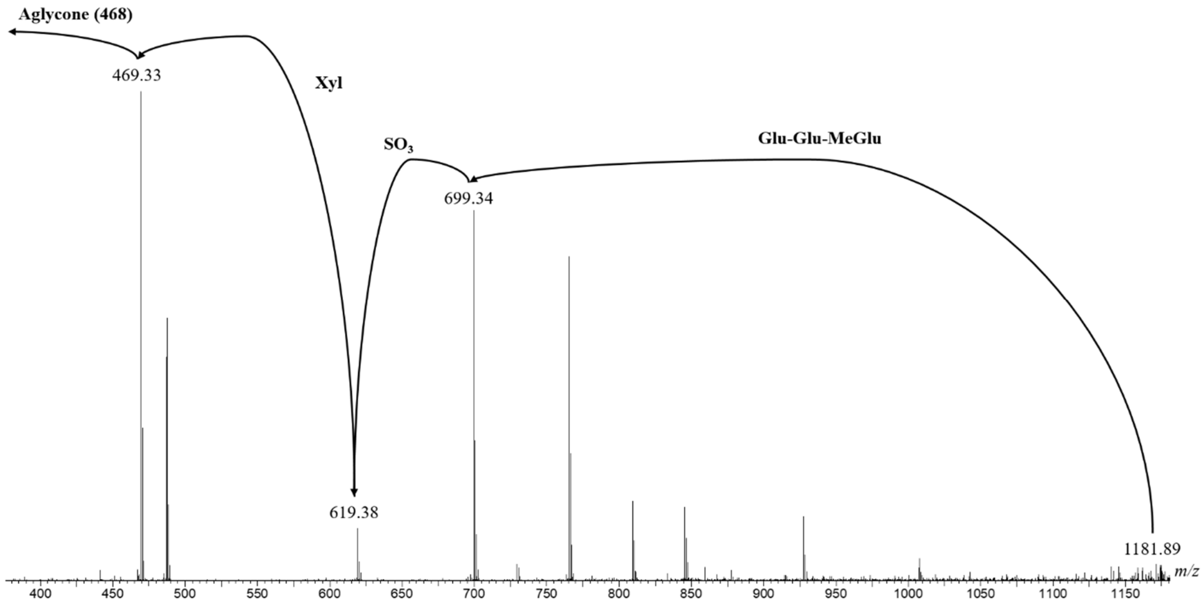

Figure S10. LC-MS/MS(+) analysis of *H.* (*H.*) *algeriensis* saponin extract: CID spectrum recorded for the *m*/*z* 1181 precursor ions $[M+H]^+$ at 8.7 min retention time (Saponin E).

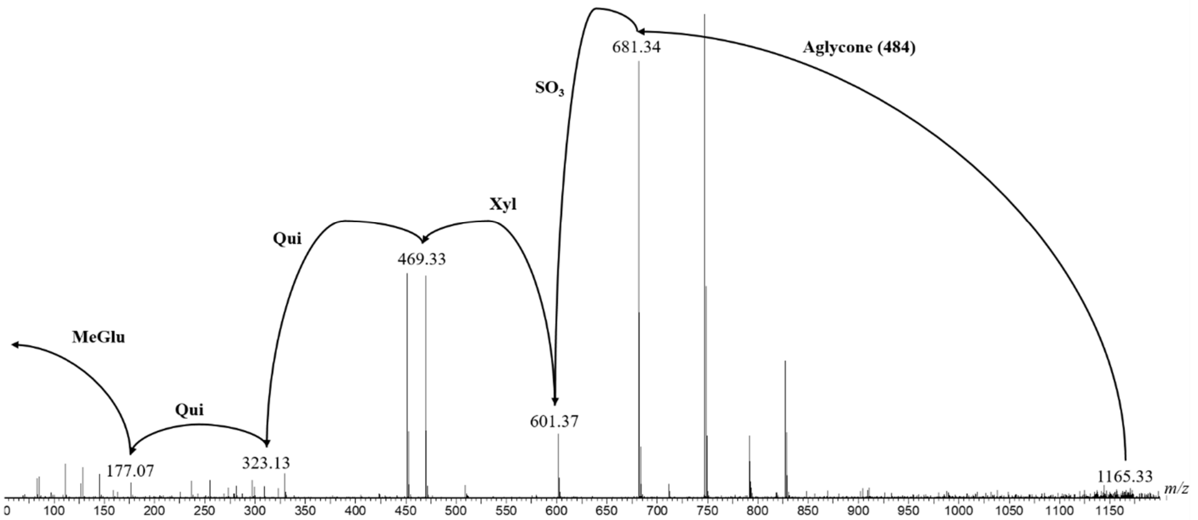

Figure S11. LC-MS/MS(+) analysis of *H.* (*H.*) *algeriensis* saponin extract: CID spectrum recorded for the *m/z* 1165 precursor ions [M+H]$^+$ at 7.9 min retention time (Saponin F).

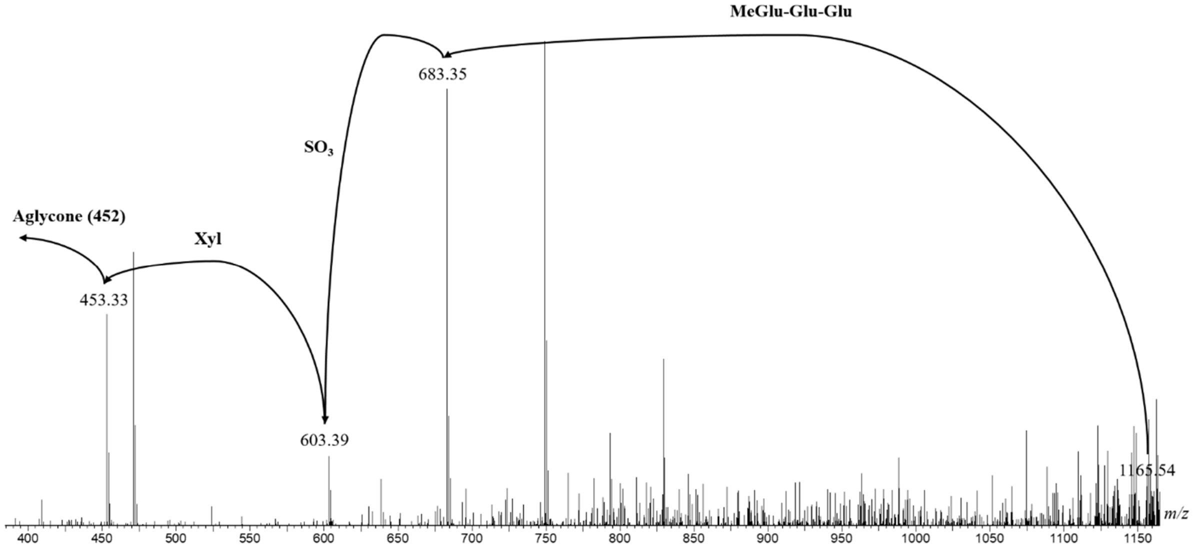

Figure S12. LC-MS/MS(+) analysis of *H.* (*H.*) *algeriensis* saponin extract: CID spectrum recorded for the m/z 1165 precursor ions [M+H]$^+$ at 11.3 min retention time (Saponin G).

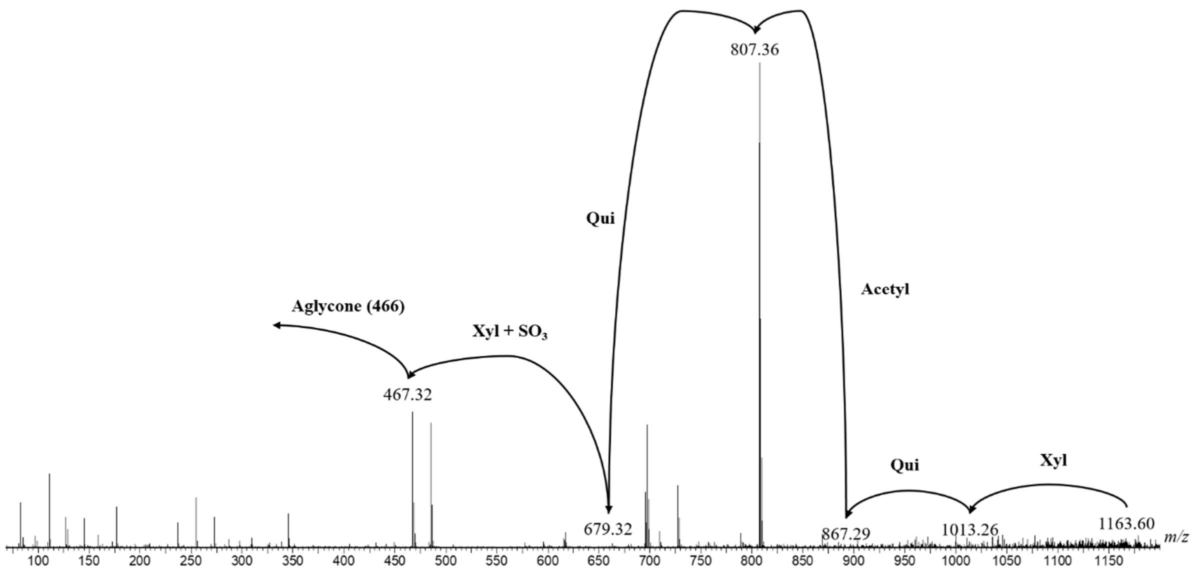

Figure S13. LC-MS/MS(+) analysis of *H.* (*H.*) *algeriensis* saponin extract: CID spectrum recorded for the *m*/*z* 1163 precursor ions $[M+H]^+$ at 7.6 min retention time (Saponin H).

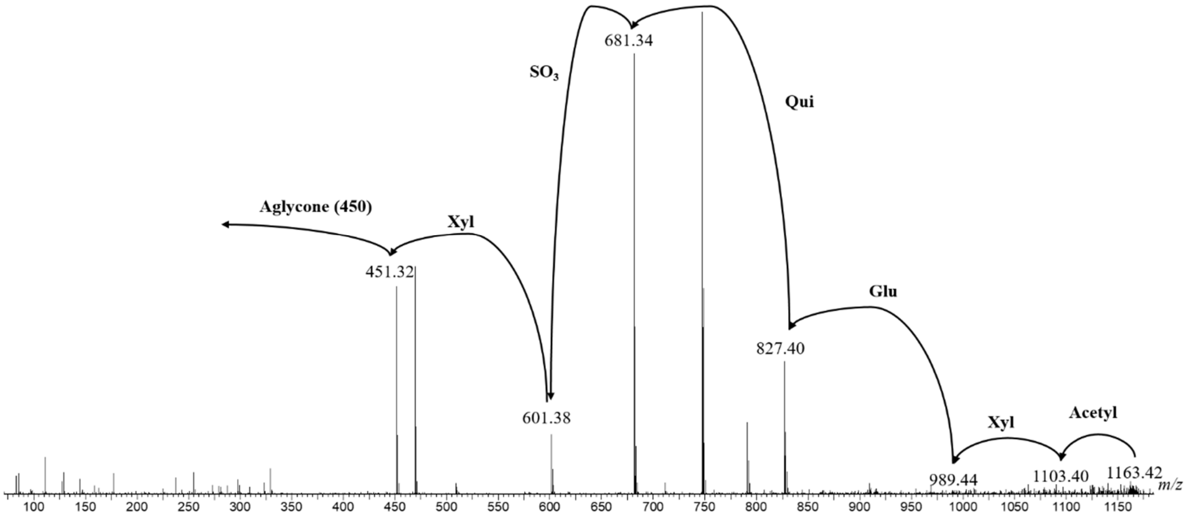

Figure S14. LC-MS/MS(+) analysis of *H.* (*H.*) *algeriensis* saponin extract: CID spectrum recorded for the *m*/*z* 1163 precursor ions $[M+H]^+$ at 7.9 min retention time (Saponin I).

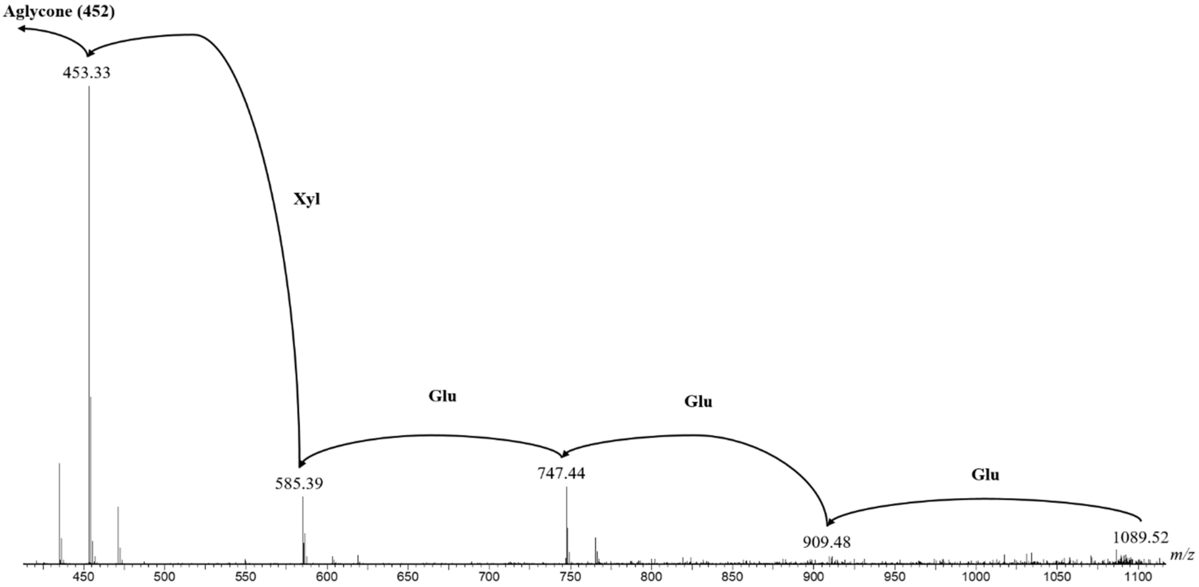

Figure S15. LC-MSMS(+) analysis of *H.* (*H.*) *algeriensis* saponin extract: CID spectrum recorded for the m/z 1089 precursor ions [M+H]$^+$ at 9.3 min retention time (Saponin J).

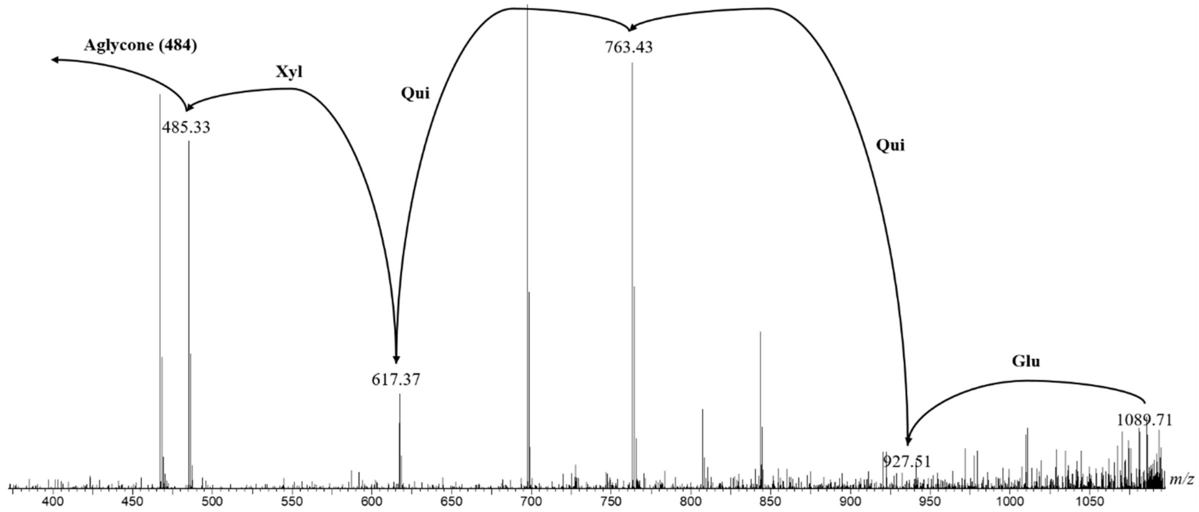

Figure S16. LC-MSMS(+) analysis of *H.* (*H.*) *algeriensis* saponin extract: CID spectrum recorded for the m/z 1089 precursor ions [M+H]$^+$ at 7.6 min retention time (Saponin K).

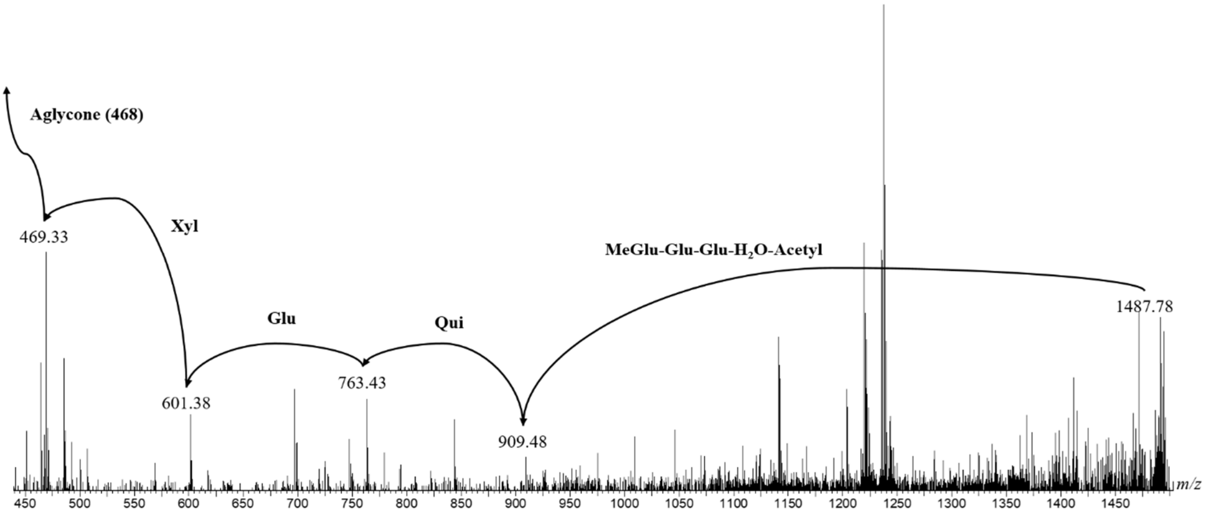

Figure S17. LC-MS/MS(+) analysis of *H.* (*R.*) *arguinensis* saponin extract: CID spectrum recorded for the *m/z* 1487 precursor ions [M+H]+ at 7.6 min retention time (Saponin 1).

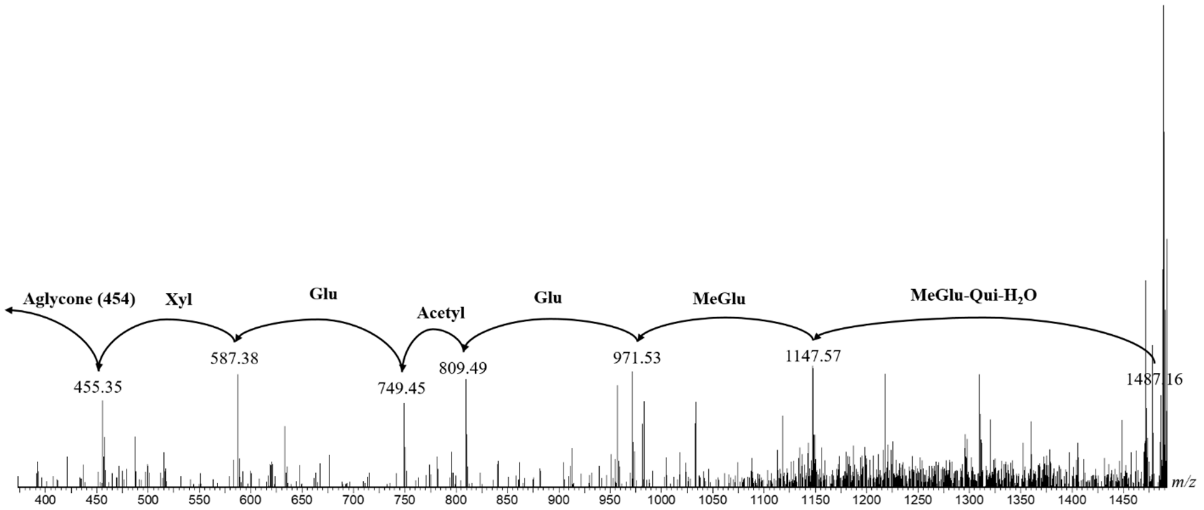

Figure S18. LC-MS/MS(+) analysis of *H.* (*R.*) *arguinensis* saponin extract: CID spectrum recorded for the *m/z* 1487 precursor ions [M+H]+ at 8.2 min retention time (Saponin 2).

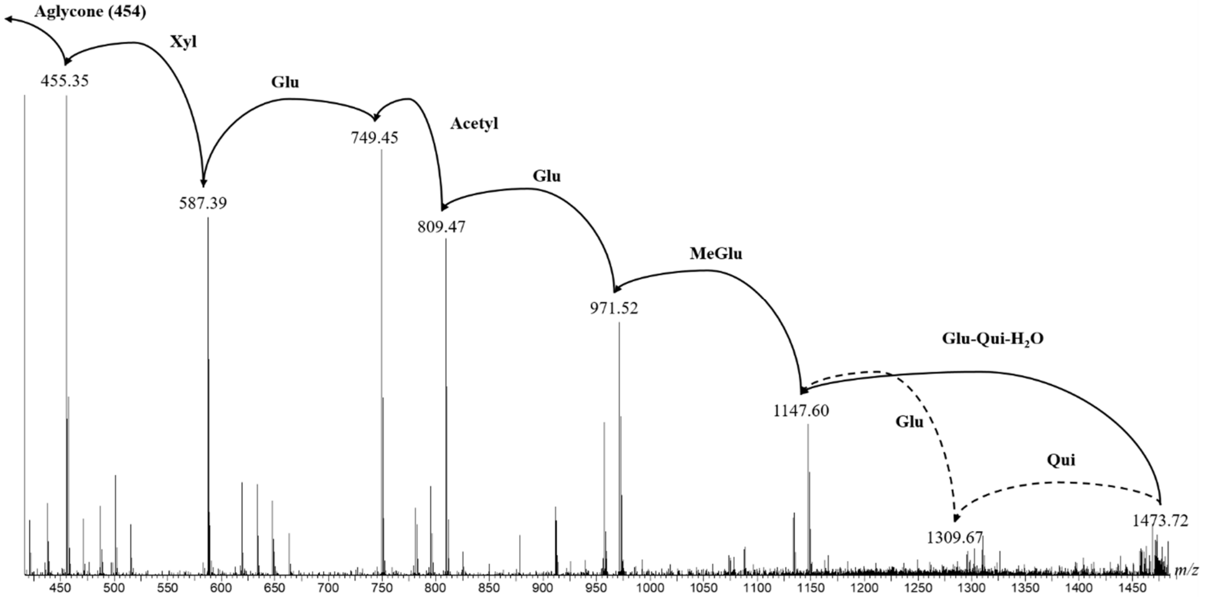

Figure S19. LC-MS/MS(+) analysis of *H.* (*R.*) *arguinensis* saponin extract: CID spectrum recorded for the *m/z* 1473 precursor ions [M+H]+ at 8.2 min retention time (Saponin 3).

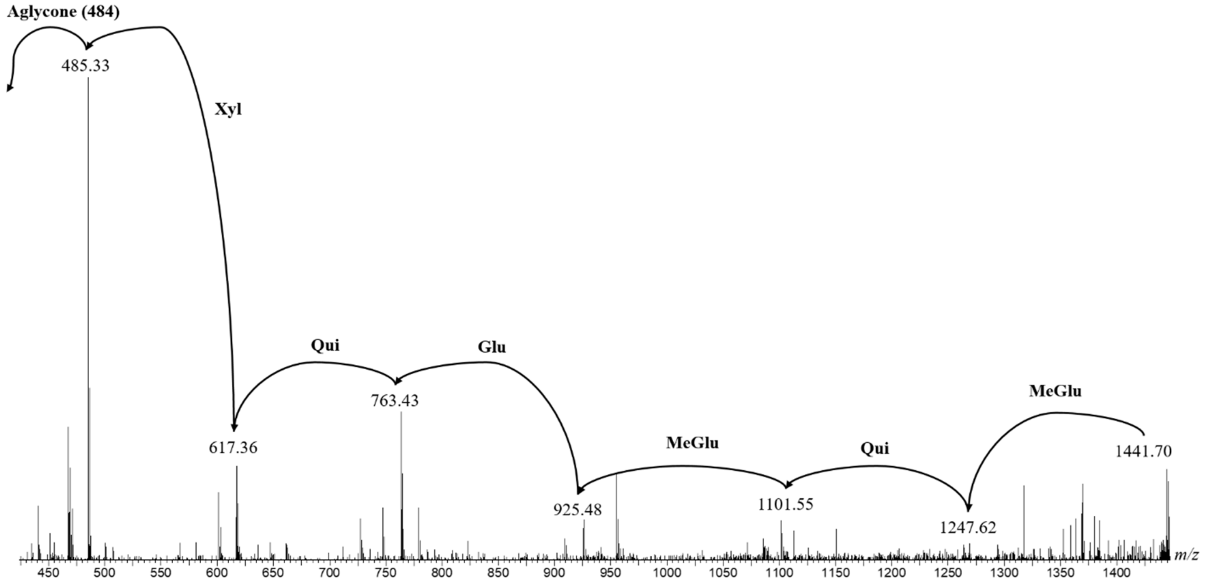

Figure S20. LC-MS/MS(+) analysis of *H.* (*R.*) *arguinensis* saponin extract: CID spectrum recorded for the *m/z* 1141 precursor ions [M+H]+ at 7.3 min retention time (Saponin 4).

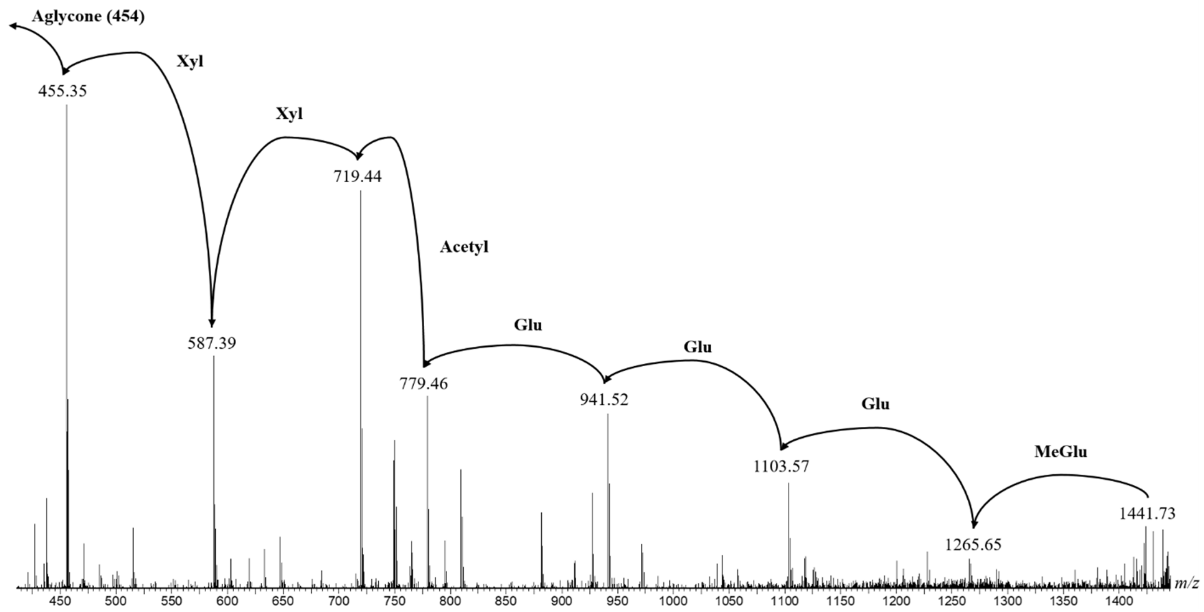

Figure S21. LC-MS/MS(+) analysis of *H.* (*R.*) *arguinensis* saponin extract: CID spectrum recorded for the *m/z* 1141 precursor ions $[M+H]^+$ at 8.4 min retention time (Saponin 5).

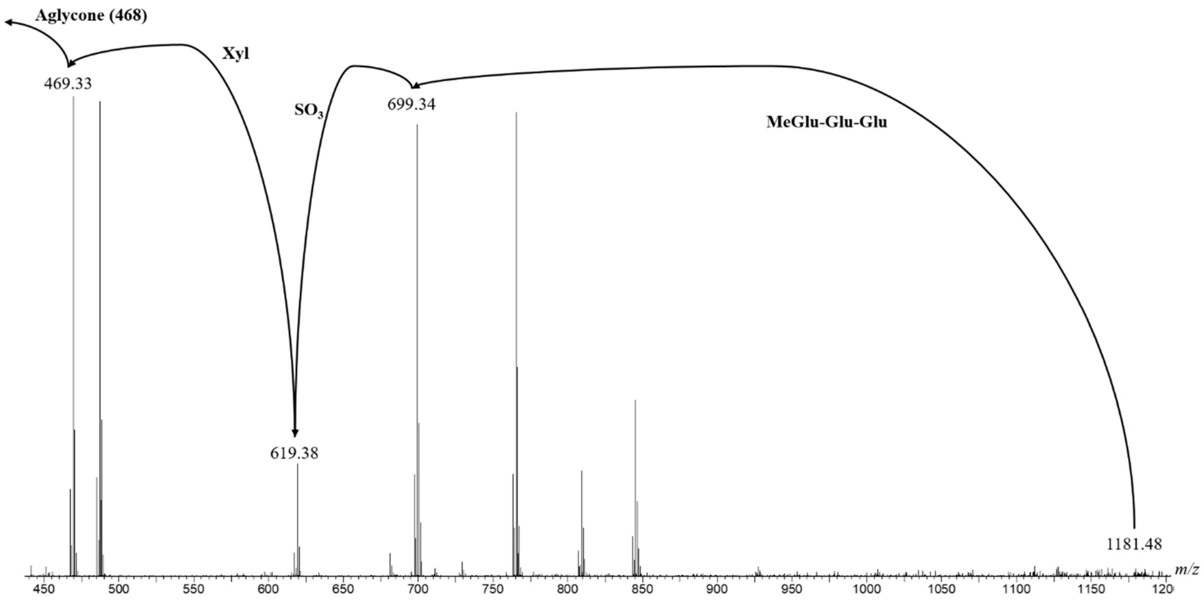

Figure S22. LC-MS/MS(+) analysis of *H.* (*R.*) *arguinensis* saponin extract: CID spectrum recorded for the *m/z* 1181 precursor ions $[M+H]^+$ at 7.4 min retention time (Saponin 6).

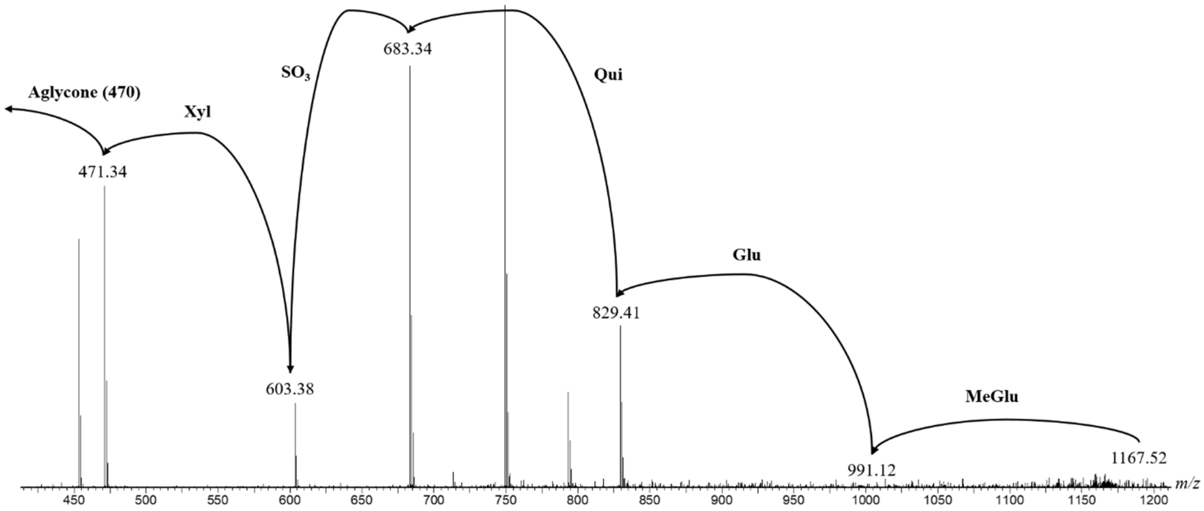

Figure S23. LC-MS/MS(+) analysis of *H.* (*R.*) *arguinensis* saponin extract: CID spectrum recorded for the *m/z* 1167 precursor ions $[M+H]^+$ at 11.3 min retention time (Saponin 7).

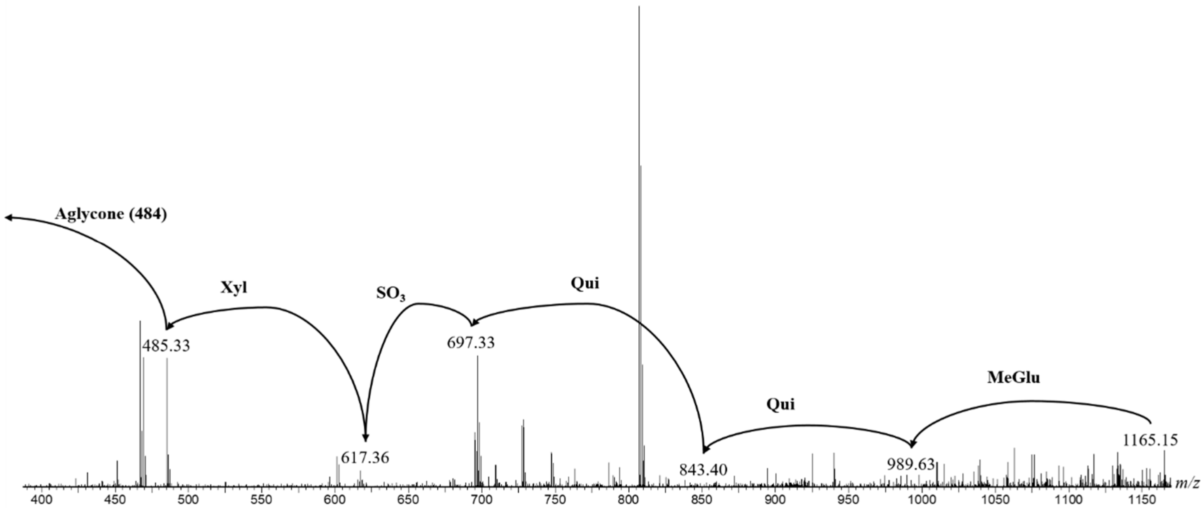

Figure S24. LC-MS/MS(+) analysis of *H.* (*R.*) *arguinensis* saponin extract: CID spectrum recorded for the *m/z* 1165 precursor ions $[M+H]^+$ at 7.6 min retention time (Saponin 8).

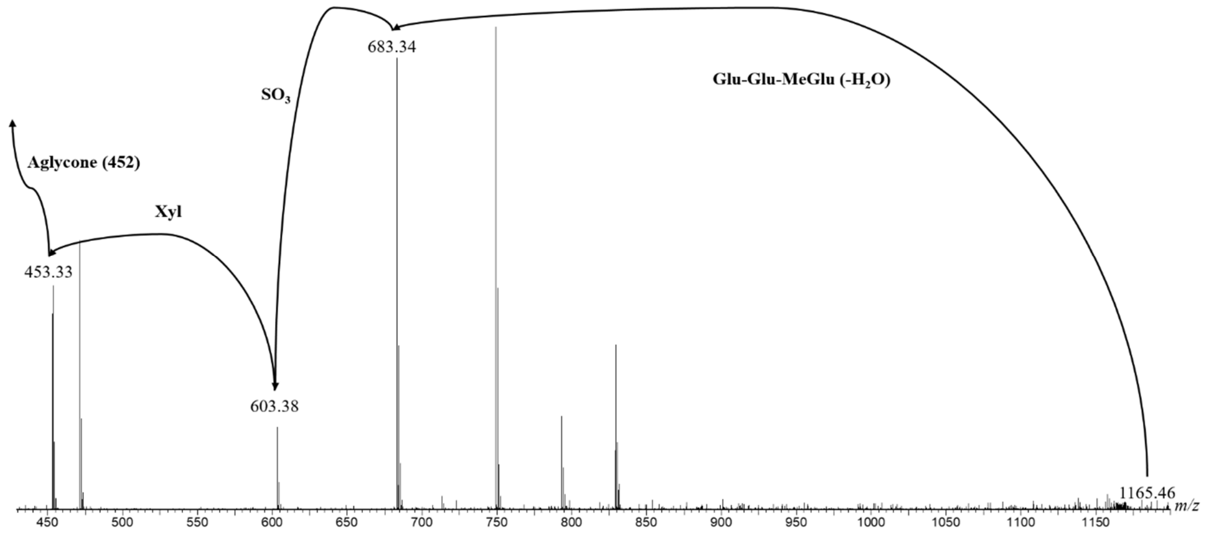

Figure S25. LC-MS/MS(+) analysis of *H.* (*R.*) *arguinensis* saponin extract: CID spectrum recorded for the *m/z* 1165 precursor ions $[M+H]^+$ at 11.3 min retention time (Saponin 9).

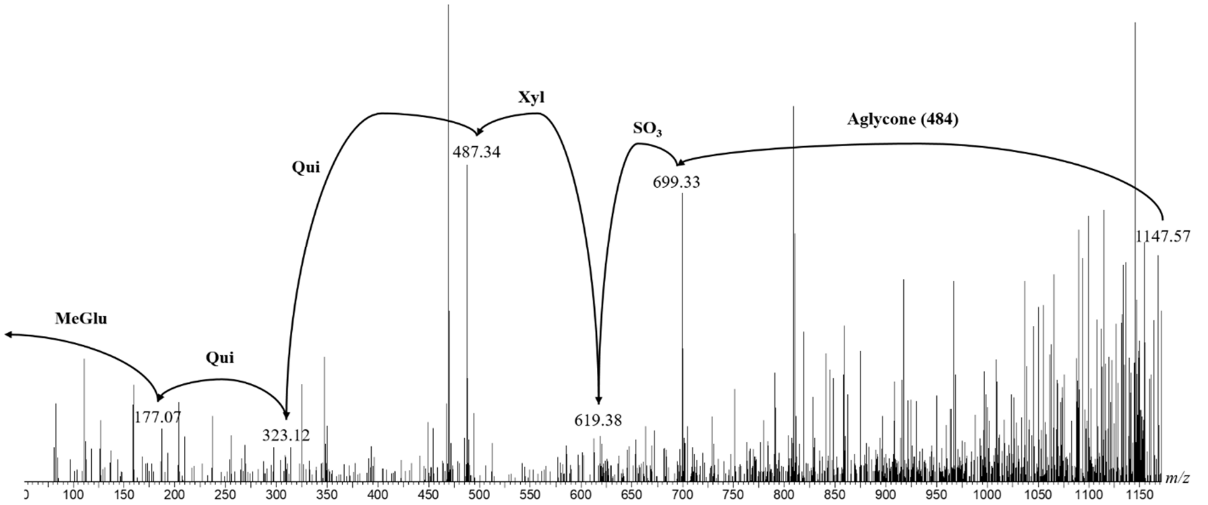

Figure S26. LC-MS/MS(+) analysis of *H.* (*R.*) *arguinensis* saponin extract: CID spectrum recorded for the *m/z* 1147 precursor ions $[M+H]^+$ at 7.4 min retention time (Saponin 10).

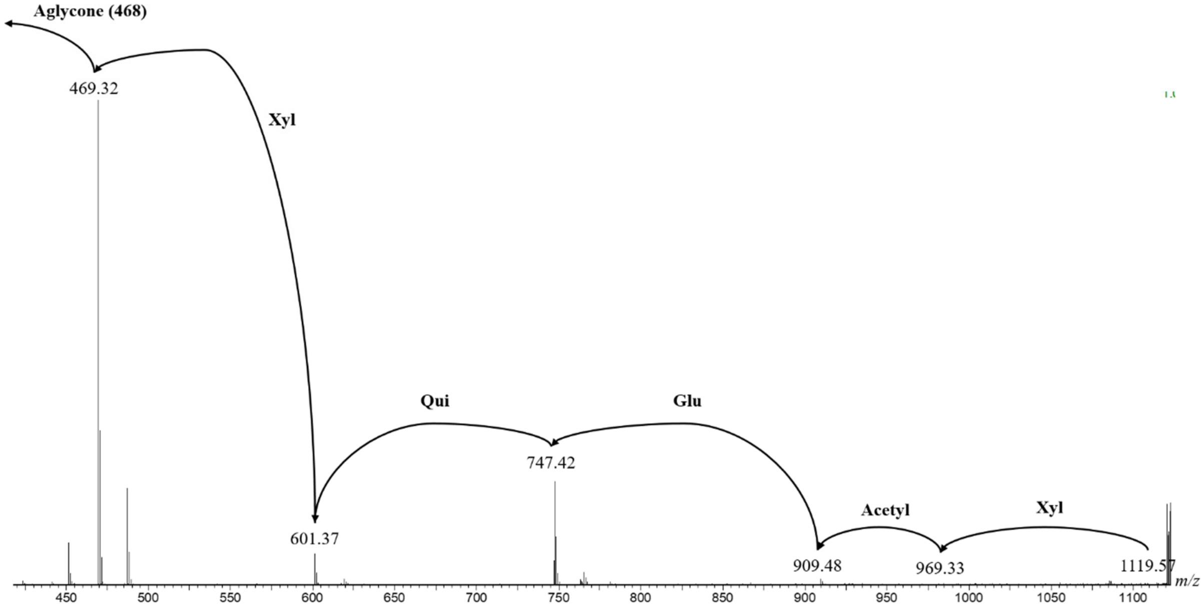

Figure S27. LC-MS/MS(+) analysis of *H.* (*R.*) *arguinensis* saponin extract: CID spectrum recorded for the *m*/*z* 1119 precursor ions $[M+H]^+$ at 10.1 min retention time (Saponin 11).

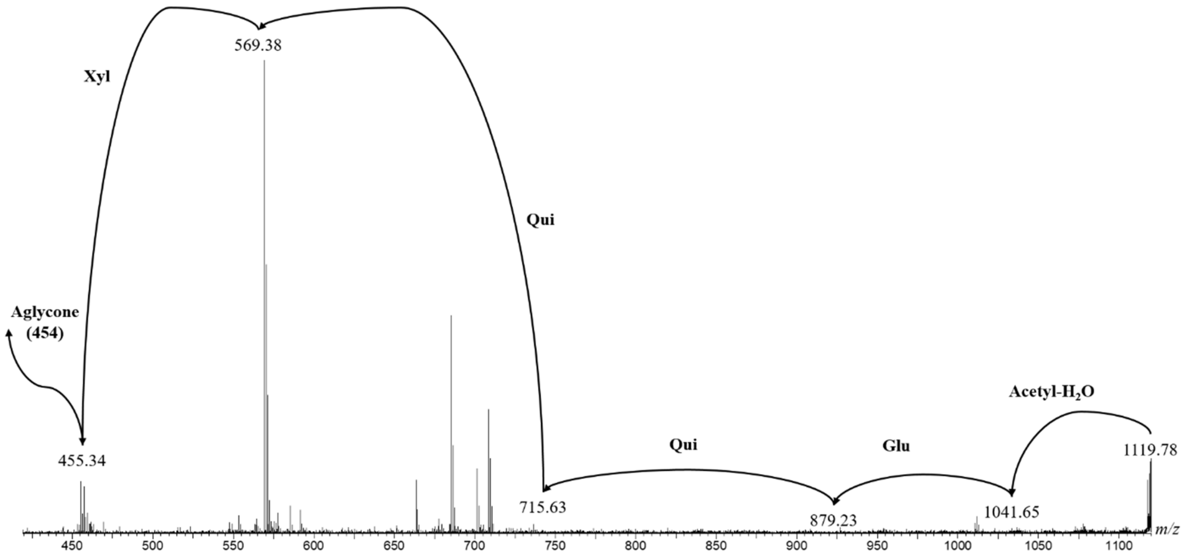

Figure S28. LC-MS/MS(+) analysis of *H.* (*R.*) *arguinensis* saponin extract: CID spectrum recorded for the *m*/*z* 1119 precursor ions $[M+H]^+$ at 13.6 min retention time (Saponin 12).

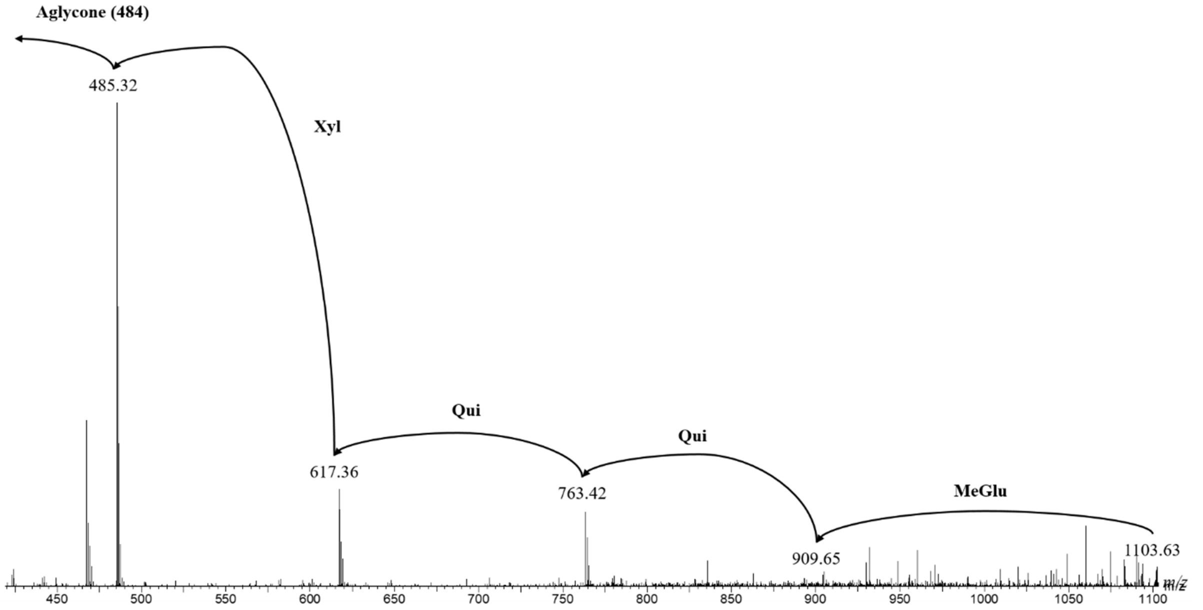

Figure S29. LC-MS/MS(+) analysis of *H.* (*R.*) *arguinensis* saponin extract: CID spectrum recorded for the *m/z* 1103 precursor ions $[M+H]^+$ at 7.6 min retention time (Saponin 13).

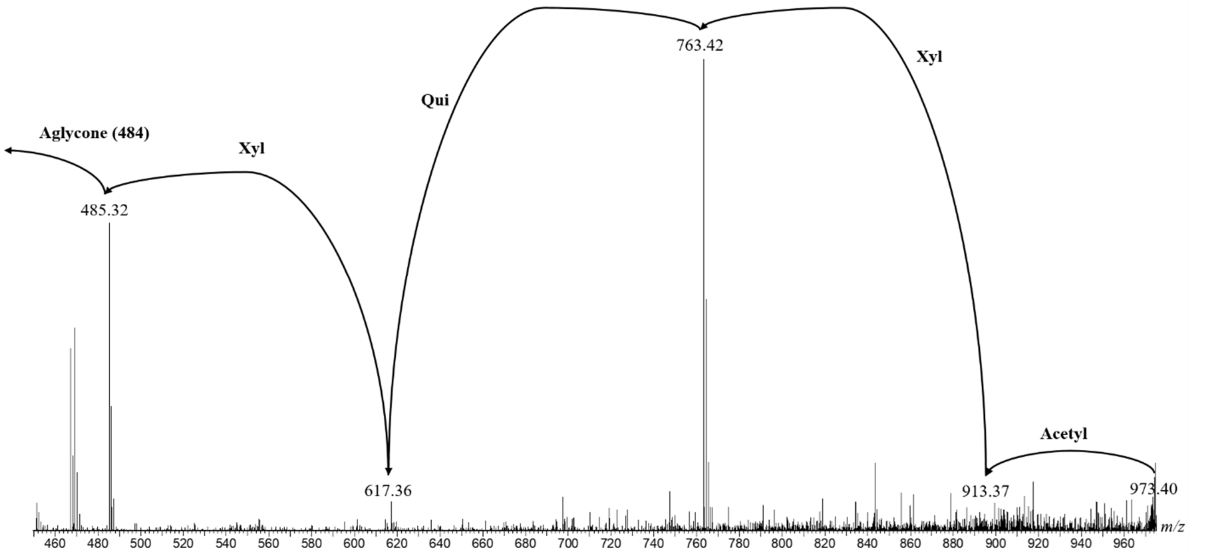

Figure S30. LC-MS/MS(+) analysis of *H.* (*R.*) *arguinensis* saponin extract: CID spectrum recorded for the *m/z* 973 precursor ions $[M+H]^+$ at 10.1 min retention time (Saponin 14).

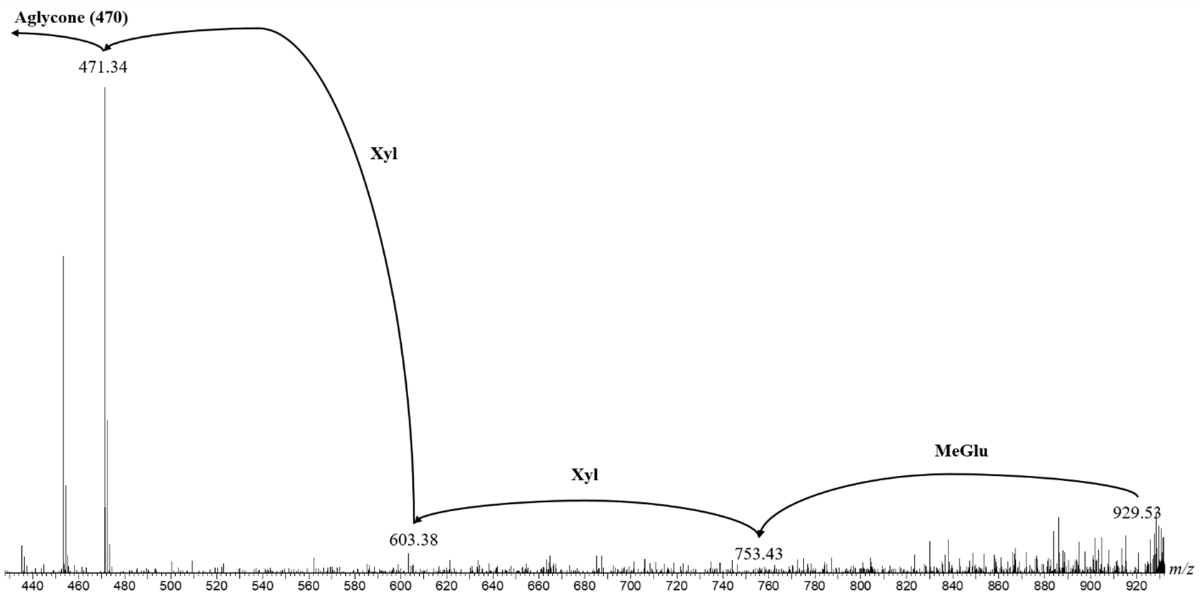

Figure S31. LC-MS/MS(+) analysis of *H.* (*R.*) *arguinensis* saponin extract: CID spectrum recorded for the *m/z* 929 precursor ions $[M+H]^+$ at 11.6 min retention time (Saponin 15).

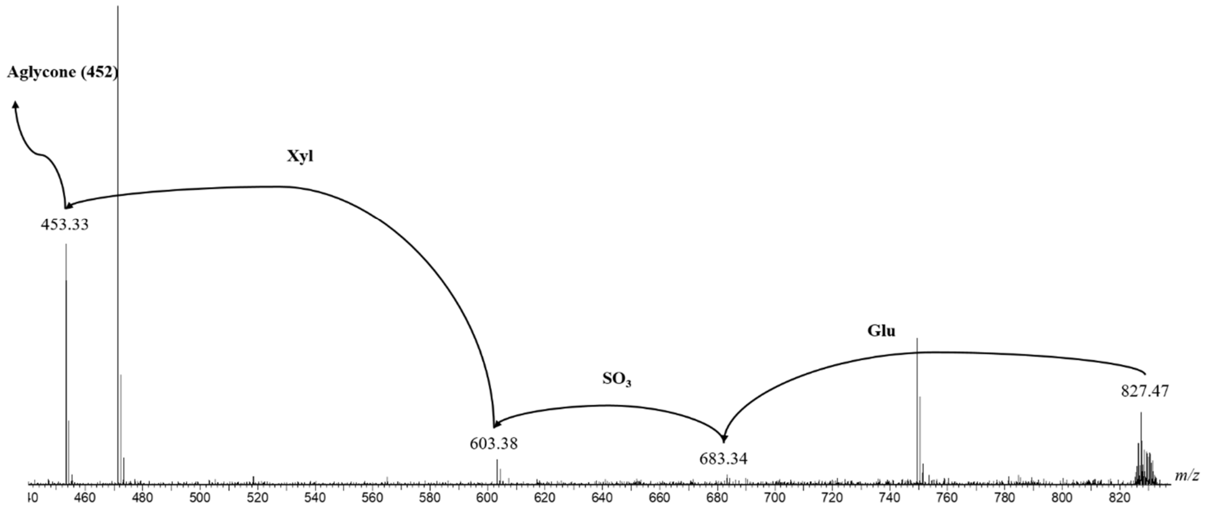

Figure S32. LC-MS/MS(+) analysis of *H.* (*R.*) *arguinensis* saponin extract: CID spectrum recorded for the *m/z* 827 precursor ions $[M+H]^+$ at 12.0 min retention time (Saponin 16).
